# Enhancing ASP Flooding by Using Special Combinations of Surfactants and Starch Nanoparticles

**DOI:** 10.3390/molecules28155770

**Published:** 2023-07-31

**Authors:** Hasanain A. Al-Jaber, Agus Arsad, Sulalit Bandyopadhyay, Muslim Abdurrahman, Mohammad Yasin Abdulfatah, Augustine Agi, Suriatie Mat Yusuf, Abdulmunem R. Abdulmunem, Muhammad Tahir, Mustafa Jawad Nuhma

**Affiliations:** 1 UTM-MPRC Institute for Oil and Gas, Faculty of Engineering, Universiti Teknologi Malaysia, Skudai, Johor 81310, Malaysia; hasanain1975@graduate.utm.my; 2 Department of Chemical Industries Technologies, Southern Technical University, Basrah 61006, Iraq; 3 Department of Chemical Engineering, Norwegian University of Science and Technology, Høgskoleringen 1, 7491 Trondheim, Norway; sulalit.bandyopadhyay@ntnu.no; 4 Department of Petroleum Engineering, Faculty of Engineering, Universitas Islam Riau, Pekanbaru 28284, Riau, Indonesia; 5 Exploration and Development Department, PT. SPR Langgak, Jakarta 12550, Indonesia; 6 Faculty of Chemical and Process Engineering Technology, College of Engineering Technology, Universiti Malaysia Pahang, Gambang 26300, Pahang, Malaysia; 7 Centre for Research in Advanced Fluid and Processes (Fluid Centre), Universiti Malaysia Pahang, Gambang 26300, Pahang, Malaysia; 8 Oil and Gas Engineering Department, College of Engineering, Universiti Technologi Mara (UiTM), Shah Alam 40450, Selangor, Malaysia; 9 Electromechanical Engineering Department, University of Technology-Iraq, Baghdad 10066, Iraq; 10 Chemical and Petroleum Engineering Department, United Arab Emirates University (UAEU), Al Ain P.O. Box 15551, United Arab Emirates; 11 Chemical Engineering Department, College of Engineering, University of Al-Qadisiyah, Al Diwaniyah City P.O. Box 88, Iraq

**Keywords:** improved ASP flooding, nano-polymer flooding, cassava nanoparticles, purple yam nanoparticles, tertiary recovery technique

## Abstract

This study aimed to address the challenges faced by mature oilfields in extracting substantial oil quantities. It focused on improving the efficiency of alkaline–surfactant–polymer (ASP) flooding technique, which is a proven tertiary recovery technology, to overcome scaling issues and other hindrances in its large-scale implementation. Appropriate materials and their suitable concentrations were selected to enhance the ASP flooding technique. Special surfactants from Indonesia were introduced to improve the interfacial tension reduction and wettability alteration. Reservoir rock model that resembling Langgak oilfield in Sumatra was utilized, and low-salinity water was employed to mimic the oilfield conditions. Starches derived from cassava nanoparticles (CSNPs) and purple yam nanoparticles (PYNPs) were combined separately with conventional hydrolyzed polyacrylamide (HPAM) polymer to enhance its performance. Sodium hydroxide and sodium carbonate were used as alkaline in final ASP formula. It was demonstrated from this research that only two combinations of ASP formulations have led to improved oil recovery. One combination utilizing PYNPs resulted in 39.17% progressive recovery, while the other combination incorporating CSNPs achieved 35% incremental oil recovery. The ASP combination that resulted in recovery rate of 39.17% was composed of sodium hydroxide (NaOH) at a concentration of 1.28 wt.%, PSC EOR 2.2 (0.98 wt.%), and a combined polymer consisting of HPAM (0.2 wt.%) and PYNPs nano-starch (0.6 wt.%). The second combination led to 35% recovery rate and involved NaOH also at concentration 1.28 wt.%, PSC HOMF (0.63 wt.%), and a combined polymer comprising from HPAM (0.2 wt.%) and CSNPs nano-starch (0.8 wt.%). These findings of this study highlighted the potential of this modified ASP flooding to enhance oil recovery in mature oilfields, thereby offering valuable insights for oil industry.

## 1. Introduction

With the depletion of oil resources, the alkaline–surfactant–polymer (ASP) flooding technique has been used, especially for reservoirs that have harsh conditions, such as high temperatures and high salinity. For such oilfields, severe conditions bring technical challenges, and, therefore, new methods for chemical flooding must be applied to overcome these challenges. Hongyan et al. noted that the field application of ASP flooding has achieved technical success, with incremental oil recovery of more than 20% [1]. The combined impact of the three elements, alkali, surfactant, and polymer, has played a significant role in effectively stimulating the remaining oil following conventional water flooding.

Substantial quantities of oil persist in the reservoir even after primary and secondary operations, necessitating the use of enhanced oil recovery (EOR) as a viable solution. In a study performed by Al-Jaber et al. [2], innovative nano-polymeric materials derived from purple yam and cassava starches were synthesized. The yield of purple yam nanoparticles (PYNPs) reached 85%, while cassava nanoparticles (CSNPs) exhibited a yield of 90.53%. Extensive characterization of the synthesized materials encompassed particle size distribution (PSA), Zeta potential distribution, Fourier transform infrared spectroscopy (FTIR), differential scanning calorimetry (DSC), and transmission electron microscopy (TEM). The recovery experiments revealed the superior performance of PYNPs in oil recovery compared to CSNPs. Zeta potential distribution results confirmed the greater stability of PYNPs (−36.3 mV) compared to CSNPs (−10.7 mV). Through interfacial tension measurements and rheological property evaluation, the optimal concentrations of these nanoparticles were determined to be 0.60 wt.% for PYNPs and 0.80 wt.% for CSNPs. The polymer containing PYNPs exhibited higher incremental recovery (33.46%) compared to the other nano-polymer (31.3%). This breakthrough offers opportunities for a novel polymer flooding technology that has the potential to replace the conventional method that is reliant on hydrolyzed polyacrylamide (HPAM).

Wang et al. [3] improved the performance of traditional weak alkaline sodium carbonate (Na_2_CO_3_) ASP flooding by combining it with silicon dioxide (SiO_2_) nanoparticles (NPs) to enhance the oil recovery. Their experimental results showed that ASP with a SiO_2_ nanoparticle mixture had good potential to reduce the interfacial tension (IFT) between the oil and water layers as well as the contact angle. The SiO_2_ NP/ASP mixture increased the oil recovery by 6.67% of the original oil in place (OOIP) in comparison to the weakly alkaline ASP solution. Despite the benefits of increased oil recovery with the application of the ASP technique, due to the limitations of applying this technique in large-scale operation, the need to find new materials such as NPs to be combined has increased [4]. Nanoparticle materials, owing to their large surface area-to-volume ratios, are widely researched for use in EOR processes. There are different opinions regarding the nature of the alkaline used in ASP formulations. Some studies have found that using strong alkalis such as caustic soda or sodium hydroxide (NaOH) can significantly improve oil recovery [5,6]. Other studies found that using a weak base such as Na_2_CO_3_ was more effective in recovering oil [7,8,9]. Yin et al. [7] performed several experiments to obtain the optimal concentration of alkali that may lead to the best results. They observed that the IFT between oil and water decreased to a low value of 1.13 mN/m when using Na_2_CO_3_ at a concentration of 1.2 wt.%. Cheraghian et al. [10] stated that the demand for NPs for use in enhanced oil recovery (EOR) is very high. Their study focused on a review of the application of NPs in flooding processes and the effect of NPs on wettability and IFT measurements.

The addition of nanoparticles to polymer solutions for EOR processes has become of interest nowadays [11,12,13,14,15,16]. However, it is important to investigate the rheology and capillary forces of the polymers containing nanoparticles to evaluate their performance during oil recovery. Hu et al. [17] studied the effect of polymer flooding with two types of nanoparticles: nano-silica (SiO_2_) and nano-aluminum oxide (Al_2_O_3_). The results obtained in their experimental work indicated that the oil recovery factors for polymer flooding, polymer–Al_2_O_3_ combination, and polymer–SiO_2_ were 58%, 63%, and 67%, respectively. Thereby, the polymer–SiO_2_ combination provided better oil recovery in comparison to the other two solutions. Moreover, the polymer–nanoparticle solution using SiO_2_ caused a dramatic decrease in residual oil saturation (Sor), whose value became 12%, in comparison to the polymer solution without nanoparticles, which had a value of 29%. This method undoubtedly provides a sustainable technology for oil recovery when applied on a large scale and could lead to advancements with increasing oil production.

Generally, polymer solutions work by increasing the viscosity of the displacing water, thereby decreasing the water/oil mobility ratio. Volumetric and displacement sweep efficiencies are positively affected by polymer flooding. Eseimokumoh et al. [18] studied the performance of cornstarch (a local polymer) to recover additional oil after conventional water flooding. This was achieved by injecting four different samples with cornstarch solutions at varying concentrations of 500, 1000, 3000, and 9000 ppm. From the results of their experiment work, it was deduced that cornstarch had the ability to recover an additional volume of oil, nearly half the volume of oil recovered during conventional water flooding (i.e., if 50% of the oil initially in place was recovered during water flooding, cornstarch could recover an additional 25% of residual oil after water flooding). It was also found that higher concentrations of cornstarch reduced the recovery factor due to polymer adsorption on the rock surfaces, which therefore altered the rock’s wettability. To reduce the adsorption effect of cornstarch, it was recommended that the concentration of cornstarch should be measured after flooding experiments to find the optimal concentration that gives minimal adsorption and to better understand the adsorption mechanism of cornstarch.

In the study conducted by Agi et al. [19], they synthesized CSNPs from plant and fruit extracts using a weak ascorbic acid. The rheological behavior of CSNP formation was compared to that of native cassava starch (CS) and a xanthan polymer. Three techniques were employed to extract oil using CSNPs, which involved weak acid hydrolysis, ultrasonic treatment, and nanoprecipitation. These techniques demonstrated satisfactory results in producing polygonal and spherical nanoparticles with an average diameter of 100 nm. The concentration, morphology, and surface charge of the solution were identified as primary factors influencing the rheology of the produced system. The study revealed that the viscosity of the NP solution increased with a larger surface area and elevated temperature for CS and CSNPs. In contrast, the viscosity of the solution decreased with an increased temperature for the xanthan polymer. In addition, the IFT between the oil and water phases decreased (which is considered favorable) with the increasing concentration of the nanofluid, the brine, and the temperature of the injector. The use of such nano-polymers also assisted in changing the wettability of sandstone at higher salinities and reservoir temperatures. As a result, the newly synthesized polymeric material was efficient in increasing the oil recovery factor by 23%.

Based on previous research, the current work aimed to investigate the performance of different types of surfactants produced by the PT SPR Langgak Company in Indonesia, which is a well-known oil company that is responsible for operating the Langgak oilfield in Riau. The crude oil is brought from this oilfield, which is located in Sumatra, in Riau. Other types of surfactants and alkalis were evaluated in this study, and the best combination that gave higher recovery was highlighted. ASP flooding experiments were performed with two nano-biopolymers, cassava nanoparticles and purple yam nanoparticles, which were mixed with HPAM. These local nano-polymers have never been used before in ASP combinations, to our knowledge, despite the fact that cassava nanoparticles have been tested before as a new biopolymer material for polymer flooding [19].

## 2. Equipment Involved in Experimental Work

The basic equipment used in the chemical flooding experiments include the following:

1. Core Samples: These were cylindrical rock samples obtained from the reservoir, representing the porous medium where the chemical flooding experiment was conducted. Core samples allow for the study of the interaction between the chemical solution and the rock matrix.
2. Confining Vessel (Core Holder): A core holder was used to hold and confine the core samples during the experiment. It provided a controlled flow path for the injection of the chemical solution and allowed for the monitoring and measurement of the flow properties.
3. Teledyne Injection Pump: An injection pump was used to deliver the chemical solution into the core sample at a controlled flow rate. It ensured the precise injection of the desired concentration of chemicals.
4. Pressure Gauges: Pressure gauges were used in the experiment to measure the pressure changes within the core sample during the chemical flooding experiment. They provide valuable data in evaluating the performance of the chemical flooding process. The data of pressure gauge measurements are used in calculating the Resistant Factor (RF) and Residual Resistant Factor (RRF).
5. Sample Collection System (Cylinder): A sample collection system was employed to collect the effluent produced from the core sample during the experiment. This allowed for an analysis of the composition and behavior of the fluid mixture after chemical injection.
6. Brookfield RST Rheometer: This was utilized to measure and analyze the properties of the chemical solution, the effluent, and other relevant parameters during the experiment.
7. Standard Electrical Oven: To replicate the conditions of the Langgak oilfield, a temperature of approximately 60 °C was maintained and applied on the core holder and core sample. The temperature was verified using a thermocouple measurement, ensuring that it remained at the desired level throughout the experiment.
8. KRUSS EasyDyne Tensiometer K20: This is a device commonly used in surface tension measurements and interfacial analysis. It is designed to determine the surface tension of liquids and the interfacial tension between immiscible liquids. It is equipped with a high-resolution camera and advanced image processing algorithms to accurately capture and analyze the shape and dimensions of liquid drops or bubbles. The instrument allows for the precise control of the temperature, ensuring that measurements can be conducted at specific temperatures to mimic real-world conditions. The instrument operates with the Wilhelmy plate method.

## 3. Results and Discussion

### 3.1. Wettability of Core Samples

**Figure 1:**
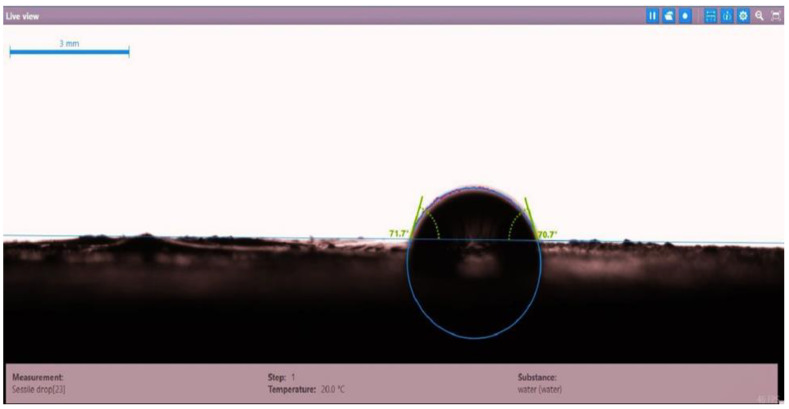
Slide of core sample (disc 1) soaked in crude oil at 60 °C.

**Figure 2:**
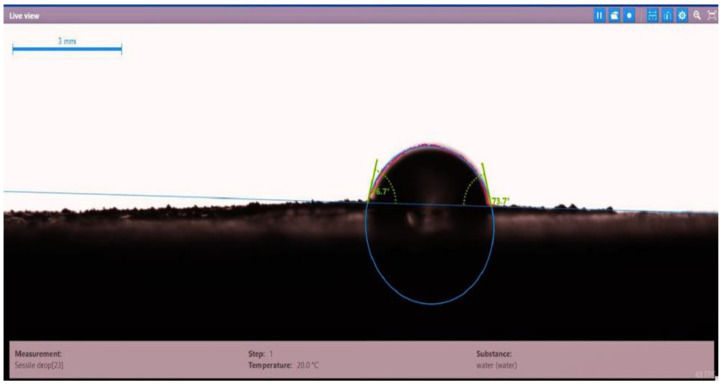
Slide of core sample (disc 2) soaked in crude oil at 60 °C.

**Figure 3:**
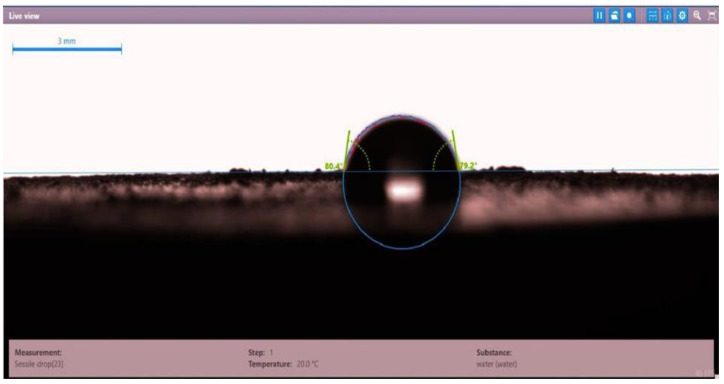
Slide of core sample (disc 3) soaked in crude oil at 60 °C.

**Figure 4:**
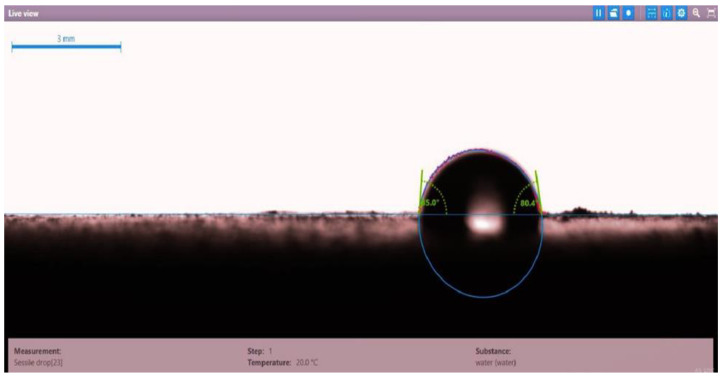
Slide of core sample (disc 4) soaked in crude oil at 60 °C.

**Figure 5:**
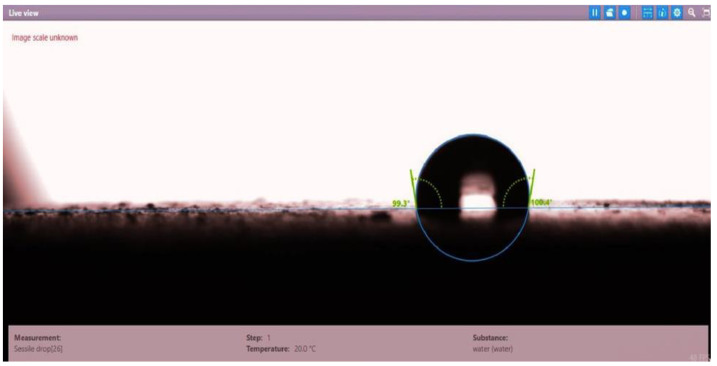
Slide of core sample (disc 5) soaked in crude oil at 60 °C.

**Figure 6:**
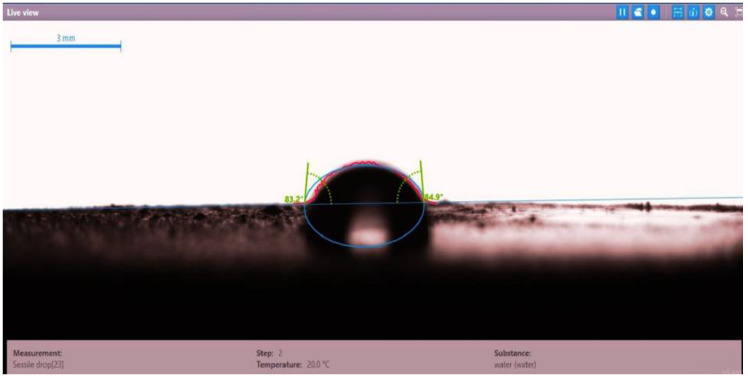
Slide of core sample (disc 6) soaked in crude oil at 60 °C.

The contact angle for six combinations was evaluated using the optical contact angle instrument located in the reservoir laboratory. Six discs were obtained by cutting one core sample, each with a thickness of 1 cm. The discs were soaked with the original crude oil after it was mixed with Fsol to ensure that it remained in a liquid state. The process of soaking took around three days at 60 °C, and then they were soaked with the prepared alkaline, surfactant, and nano-polymer combinations for another three days. The oil-wet condition was achieved when the discs were immersed with crude oil, which is the normal condition for the sandstone in the Langgak oilfield in Sumatra. When the same discs were separately soaked with different solutions at 60 °C, the water-wet condition was noticed, which is considered beneficial for oil recovery. It was observed that some solutions exhibited a good reduction in the contact angle, which indicated that this solution was considered successful, while other combinations were found to be less affected by the reduction in the contact angle. Table 1 and Table 2 demonstrate the measured values of the contact angle for these six discs. Images of the contact angles for the six discs saturated with crude oil are shown in Figure 1, Figure 2, Figure 3, Figure 4, Figure 5 and Figure 6. In these figures, the optical instrument connected to a desktop computer was used to determine the contact angle between the oil layer covering the disc’s surface area and a droplet of distilled water injected from a needle. The contact angle was measured from both the left and right sides of the droplet to provide accurate results.

**Figure 7:**
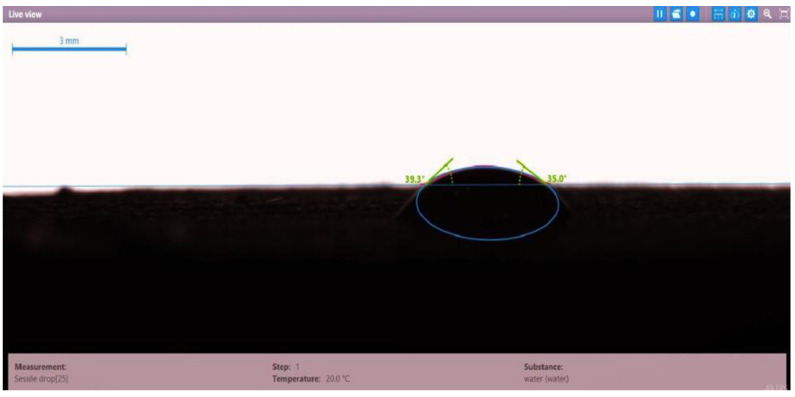
Contact angle for a disc of a core sample soaked in crude oil and PSC HOMF (0.63 wt.%) at 60 °C.

**Figure 8:**
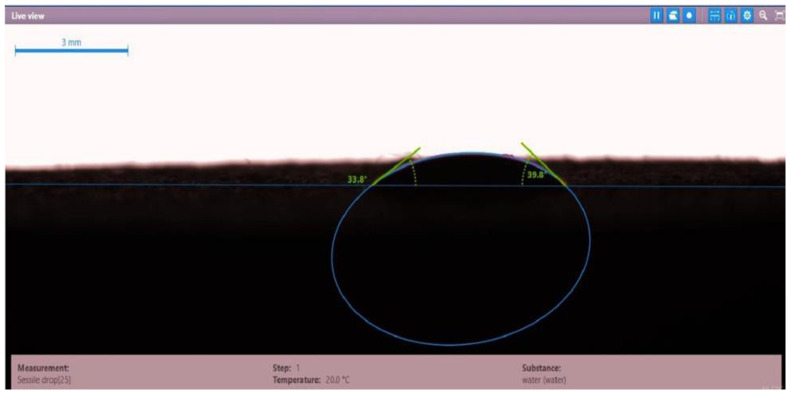
Contact angle for a disc of a core sample soaked in crude oil and Dekasurf SF 9136 (1.24 wt.%) at 60 °C.

**Figure 9:**
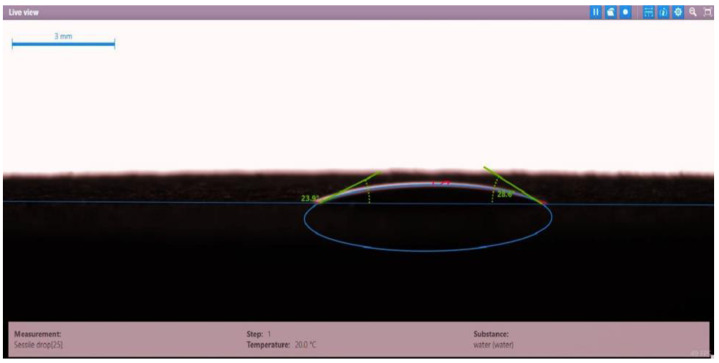
Contact angle for a disc of a core sample soaked in crude oil and Mits-5L001 (1 wt.%) at 60 °C.

**Figure 10:**
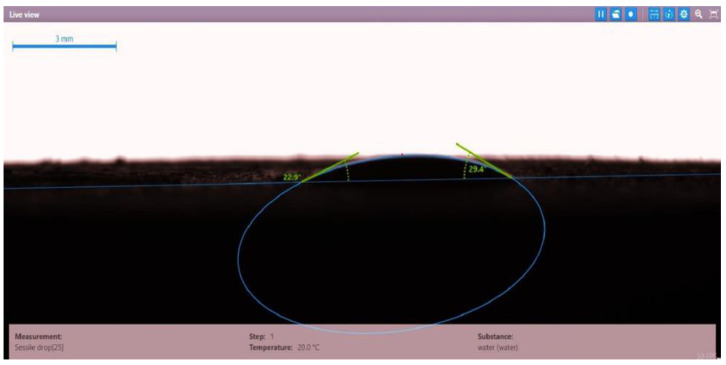
Contact angle for a disc of a core sample soaked in crude oil and PSC EOR 2.2 (0.98 wt.%) at 60 °C.

**Figure 11:**
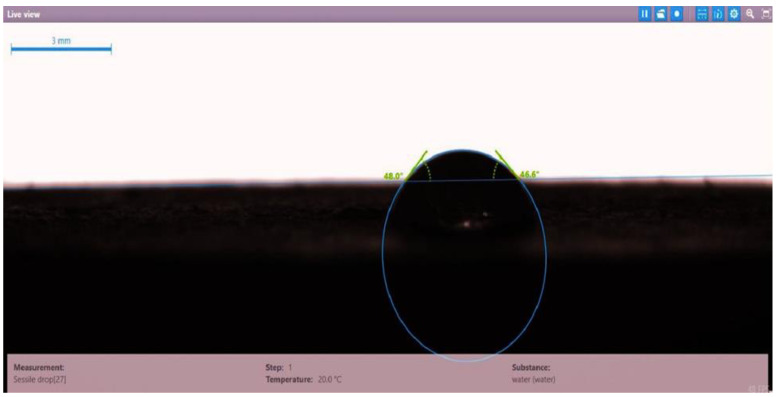
Contact angle for a disc of a core sample soaked in crude oil and HPAM (2000 ppm) + PYNPs (1.25 wt.%) at 60 °C.

**Figure 12:**
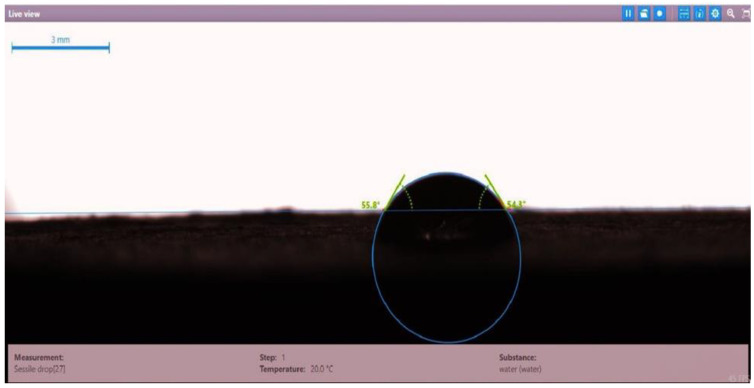
Contact angle for a disc of a core sample soaked in crude oil and HPAM (2000 ppm) + CSNPs (1.25 wt.%) at 60 °C.

As seen in Table 2, all surfactants obtained from the PT SPR Langgak Company were considered satisfactory as they significantly reduced the contact angle with crude oil from around 80° to 30°. This means that the inspected surfactants changed the wettability condition from oil-wet to water-wet, which is regarded as favorable in oil recovery. However, the nano-polymer combinations (HPAM + PYNPs or CSNPs) were found not to be satisfactory if they were applied alone in the injection without surfactants and/or alkalis. This was seen in the high value of the contact angle, which was 47.3° for the first nano-polymer, which was composed of HPAM and PYNPs (1.25 wt.%), and 55.05° for the second nano-polymer. In other words, both combinations were found to be inefficient in changing the wettability condition of the crude oil from oil-wet to water-wet. Images of the contact angles for a core sample immersed in crude oil and different combinations of surfactants/nano-polymers are shown in Figure 7, Figure 8, Figure 9, Figure 10, Figure 11 and Figure 12.

### 3.2. Compatibility of Surfactants

#### 3.2.1. Sodium Dodecyl Sulfate (SDS) Solution

**Figure 13:**
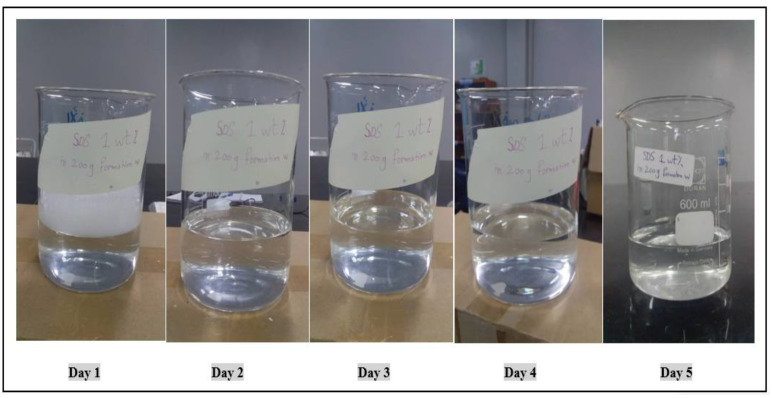
Effect of temperature on the stability of SDS at 0.3 wt.% and temperature of 60 °C.

Generally, it was found that an ultra-low IFT could be obtained when the surfactant concentration was below 0.05 wt.% and even at concentrations below 0.01 wt.% when mixtures of certain surfactants were used together at a proper ratio [20]. It could be observed from the images taken over five days that SDS was stable at the reservoir temperature and there was no phase change or change in color after it was placed in the oven for five days. The concentration set for this surfactant was 0.3 wt.%, and the surfactant was prepared with salinity of 100 ppm to simulate the original reservoir. Figure 13 shows the results of the compatibility test for this surfactant.

#### 3.2.2. Sodium Dodecylbenzene Sulfonate (SDBS) Solution

**Figure 14:**
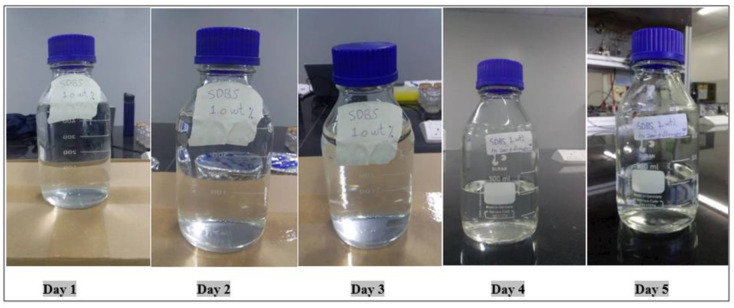
Effect of temperature on the stability of SDBS at 0.3 wt.% and 60 °C.

SDBS acts as an anionic surfactant and has been tested before in EOR processes. The interfacial and thermodynamic properties of this surfactant can be improved when it is mixed with alkaline and/or other components [21]. Moreover, this surfactant was prepared at 0.3 wt.% using brine water (100 ppm) and kept at a reservoir temperature of 60 °C. SDBS, as seen in the images, maintained its uniformity and acted as a single phase from the first day to the fifth day. Figure 14 shows the images taken for this surfactant during the 5-day period.

#### 3.2.3. Mits-5L001 Solution

**Figure 15:**
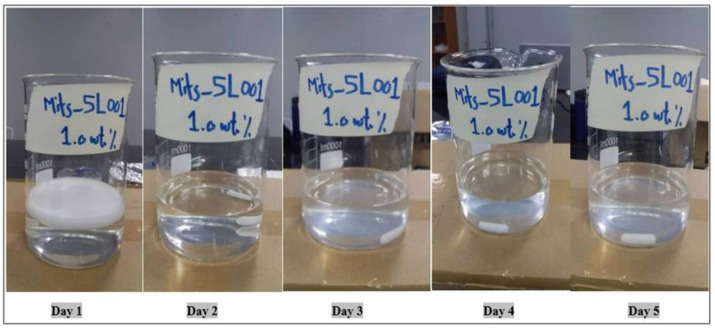
Effect of temperature on the stability of 1 wt.% Mits-5L001 at 60 °C.

The Mits-5L001 solution was manufactured by Mits Duta Utama located in Cikarang Utara, Bekasi Regency in Indonesia. It was a clear liquid with a yellowish color, and its viscosity was less than 50 cp. It was considered non-flammable and stable under normal conditions. The suitability of this surfactant was confirmed by the images taken during the five days of observation under reservoir conditions (60 °C). Moreover, the flash temperature for Mits-5L001 is around 91 °C, which makes it more resistant to harsh conditions. The yellowish color of its original solution disappeared during the preparation process as a result of dilution with brine water (100 ppm). Thus, it appeared colorless, as seen in the captured images. Figure 15 shows the effect of the temperature on the stability of this surfactant.

#### 3.2.4. PCM^TM^ HOMF Solution

**Figure 16:**
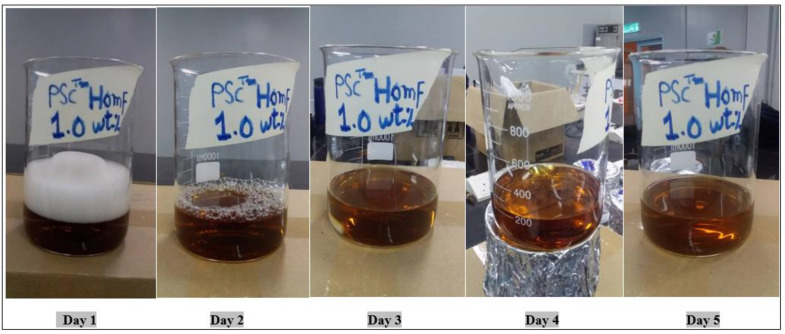
Effect of temperature on the stability of 1 wt.% PCM^TM^ HOMF at 60 °C.

PCM^TM^ HOMF is a proprietary liquid mixture produced for the purpose of extracting oil from the Langgak oilfield in Indonesia. Its proprietary blend is from 50 to 60 wt.%. This surfactant is considered concentrated, and it was diluted with water (salinity 100 ppm) before being examined for compatibility. It was diluted to a 1 wt.% concentration, and, according to the image taken on the first day, some foam was noticed, which resulted from the continuous stirring during the preparation process. However, this foam decreased significantly on day 2 and completely disappeared over the following days. The color of this surfactant was brown, and its intensity did not decrease with increasing time from day 2 to day 5 after being kept at 60 °C for five days. This indicates that it is a good candidate for flooding experiments, especially when mixed with other components. Figure 16 shows the effect of temperature on its stability over five days.

#### 3.2.5. Dekasurf SF 9136 Solution

**Figure 17:**
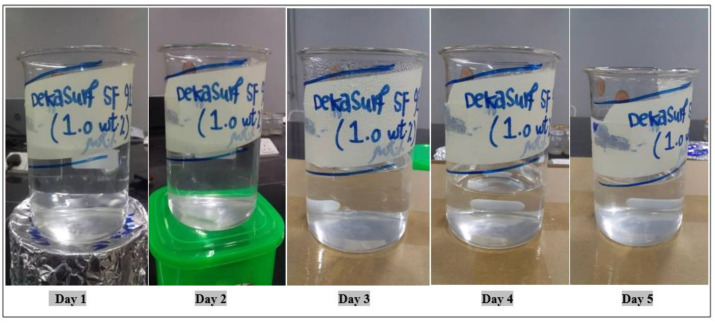
Effect of temperature on the stability of 1 wt.% Dekasurf SF 9136 at 60 °C.

The proprietary blend for the surfactant was within 50–60% by weight. One percent by weight (10,000 ppm) of this surfactant was prepared by mixing it with brine water that had salinity of 100 ppm. Images captured during the five-day period demonstrated that this surfactant was steady and stable at this concentration, with no sedimentation or stability deterioration over time. However, this product seemed to be more affected by the temperature, especially on the fourth and fifth days, as its quantity was slightly decreased. This indicates that it is a poor candidate for flooding experiments, especially at higher temperatures. Thus, other tests with this surfactant must be performed before deciding whether it is suitable for the injection process or not. Figure 17 shows the effect of the temperature on the stability of this surfactant.

#### 3.2.6. Proprietary Solution PSC EOR 2.2

**Figure 18:**
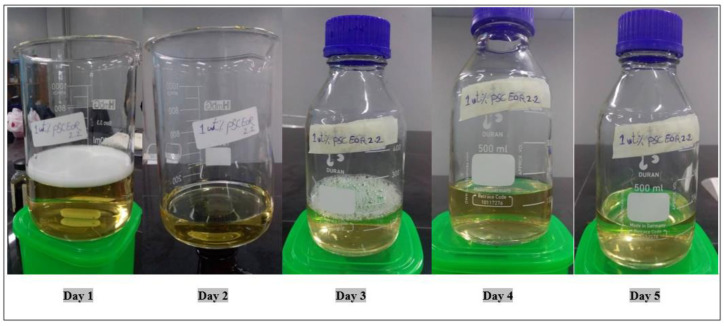
Effect of temperature on the stability of PSC EOR 2.2 (1 wt.%) at 60 °C.

Proprietary Solution PSC EOR 2.2 was analyzed as a surfactant and was obtained from the PT SPR Langgak Company in Indonesia. It is used, as mentioned in the safety data sheet, in brackish water systems. The compatibility test for this mixture was performed at a concentration of 1 wt.%, using brine water prepared at salinity of 100 ppm. The results for this test showed a clear and stable solution at a temperature of 60 °C. The yellowish color of this mixture resulted from the mixing of this surfactant (which was originally dark yellow) with the brine water. Figure 18 shows the result of the compatibility test for this surfactant at 60 °C.

### 3.3. Viscosity Stability and Surface Tension Measurements

**Figure 19:**
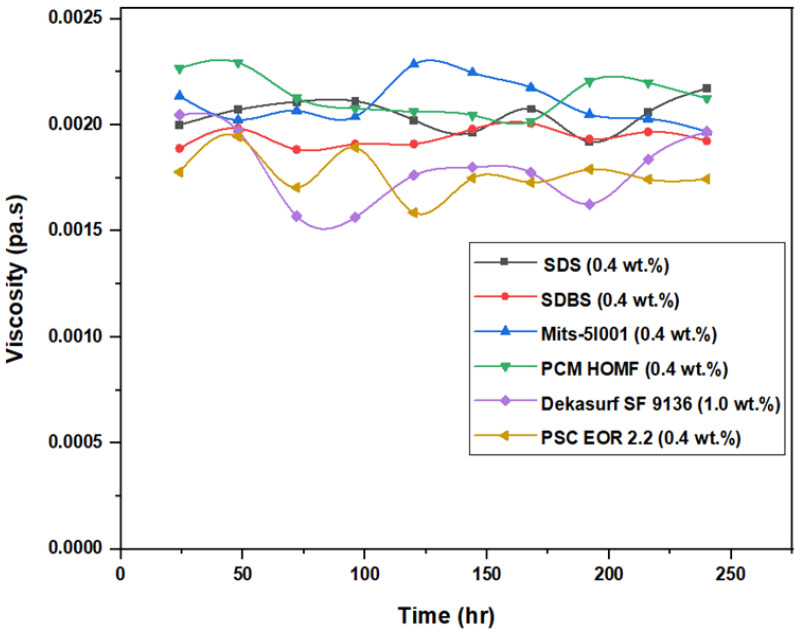
Relation between viscosity of tested surfactants and time at 60 °C.

Figure 19 illustrates the measured values of viscosity against time for all surfactants for a time frame of 10 days. For SDS, the viscosity slightly changed from 0.002 Pa.s on the first day to around 0.00206 Pa.s on the ninth day. This variation was considered normal as long as the viscosity did not change significantly during this period [22]. On the tenth day, the value of viscosity was high (0.002172 Pa.s) in comparison to the previous one. This happened due to the continuous exposure to heating during this period (10 days). The heat of the oven caused the slight evaporation of the SDS solution; therefore, the concentration of SDS was slightly increased, which affected the value of the viscosity at this time. In the same regard, the change in surface tension for SDS (0.4 wt.%) versus time was well recognized. On days 4 and 5, there was no change in the value of the surface tension (SFT), which remained constant at 40 mN/m. This indicated that, over the period of five days, there was a noticeable variation that occurred in the value of SFT for this surfactant, with a slight difference between day 2 (37 mN/m) and day 3 (42 mN/m). This led to the conclusion that this surfactant is not suitable for ASP flooding, despite the fact that it has been suggested for other EOR techniques by many researchers [22,23].

**Figure 20:**
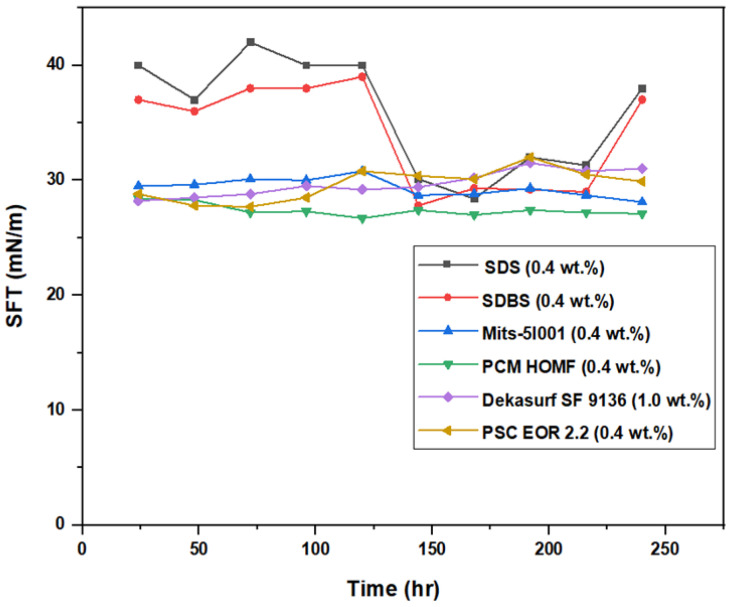
Relation between SFT for tested surfactants and time at 60 °C.

The viscosity changes for the SDBS surfactant (at 0.4 wt.%) were slightly greater than those for SDS. However, this variance was small and began within the first day of measurement at a viscosity of 0.00189 Pa.s, and it settled on the seventh day at a value of 0.002008 Pa.s. SFT measurements appeared more stable and consistent for all other surfactants, as shown in Figure 20, except for SDS and SDBS. Nevertheless, there was slight variance recorded from the sixth day to the ninth day for the SDBS surfactant, which averaged at around 29 mN/m. There was significant variation in the values of SFT for both SDS and SDBS, while the others were more stable. This test indicated that both SDS and SDBS were not satisfactory, and therefore they were both excluded from the final ASP experiments.

### 3.4. Adsorption and Injectivity Evaluation

In the current work, dynamic adsorption experiments were performed to evaluate the suitability of the surfactants and polymers for injectivity in order to construct the final combinations to be used in the ASP injectant. Surfactant consumption inside reservoirs and polymer mechanical degradation have a significant impact on the effectiveness of injected ASP slugs in recovering oil after water flooding [24]. Aqueous solutions consisting of different types of surfactants and also cassava/purple yam nanoparticles with HPAM were injected into cleaned core samples at a reservoir temperature of 60 °C. The concentrations of the surfactants and polymers in the effluent stream were measured using an ultraviolet–visible (UV–VIS) spectrophotometer, to estimate the chemical adsorption in the core samples. Dynamic adsorption experiments revealed that surfactant adsorption and polymer consumption were reduced in the presence of starch nanoparticles. They achieved favorable mobility control for ASP formation.

The results of the adsorption and injectivity tests for the surfactants and nano-polymers are shown in Table 3. The criteria for successful application were an RF and RFF less than 1.2 and an adsorption value for every tested solution of less than 0.4 mg/1g of the core weight [23]. The RF and RFF were calculated using Equations (1) and (2), respectively. As specified in the following table, the nano-polymer combination that consisted of HPAM (1000 ppm) and PYNPs (1.25 wt.%) exhibited good performance for adsorption as both RF and RFF were below 1.2. The same conclusion was reached regarding the other nano-polymer, which contained 1.25 wt.% cassava nanoparticles. For the SDS and SDBS surfactants, both RF and RFF were above 1.2, and the adsorption rate was above the recommended value (0.4 mg/g), as it was 3.25 for SDS and 3 for SDBS. This was in line with the rheology test previously performed for these two surfactants. Significant variance in viscosity was recorded during the predefined period; this gave further confirmation that these surfactants were not suitable for the final ASP flooding.

**Figure 21:**
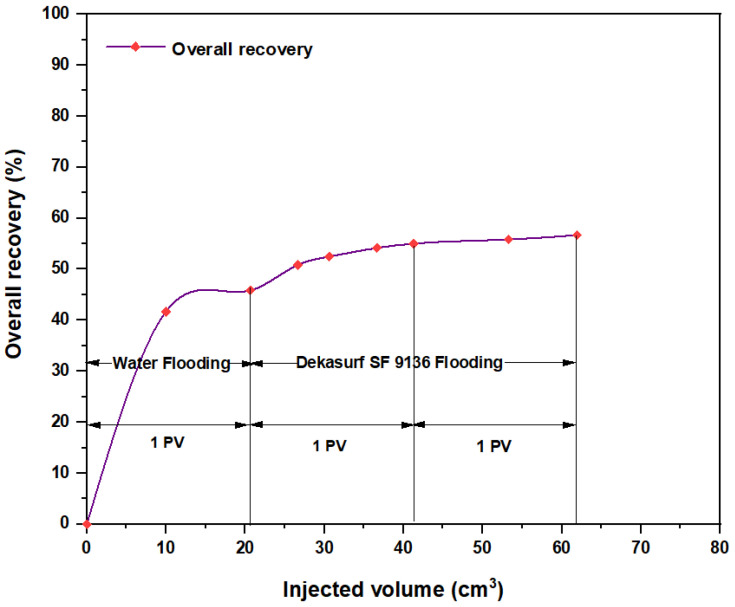
Oil extracted after water and surfactant flooding at 60 °C. The surfactant solution consisted of Dekasurf SF 9136 at 1.24 wt.%.

**Figure 22:**
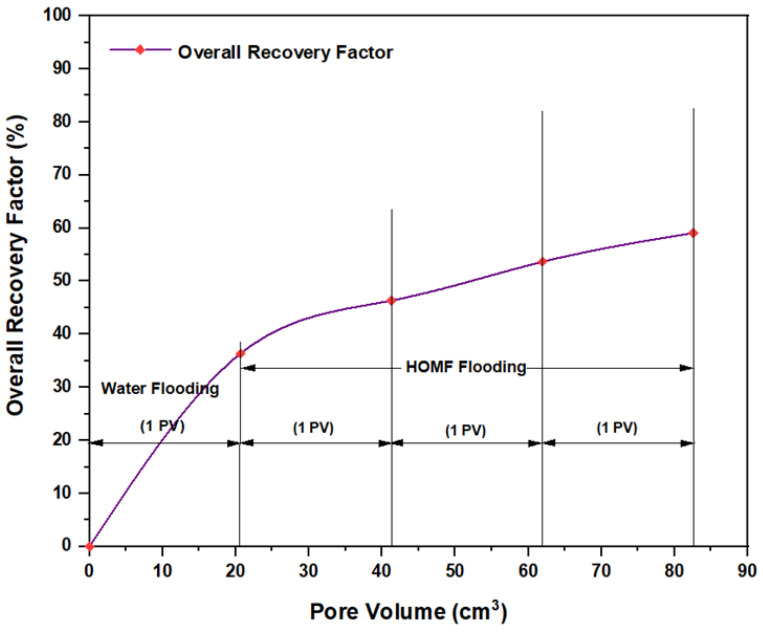
Oil extracted after water and surfactant flooding at 60 °C. The surfactant solution consisted of PSC HOMF at 0.63 wt.%.

**Figure 23:**
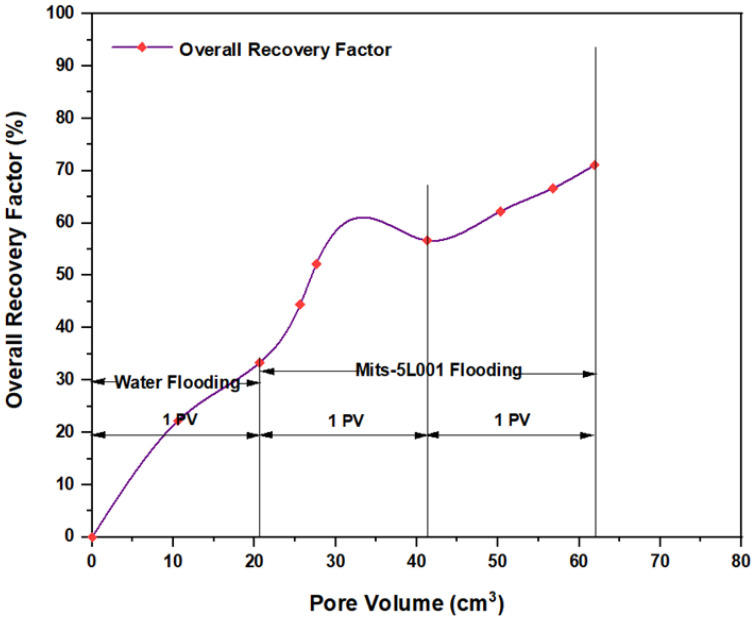
Oil extracted after water and surfactant flooding at 60 °C. The surfactant solution consisted of Mits-5L001 at 1 wt.%.

**Figure 24:**
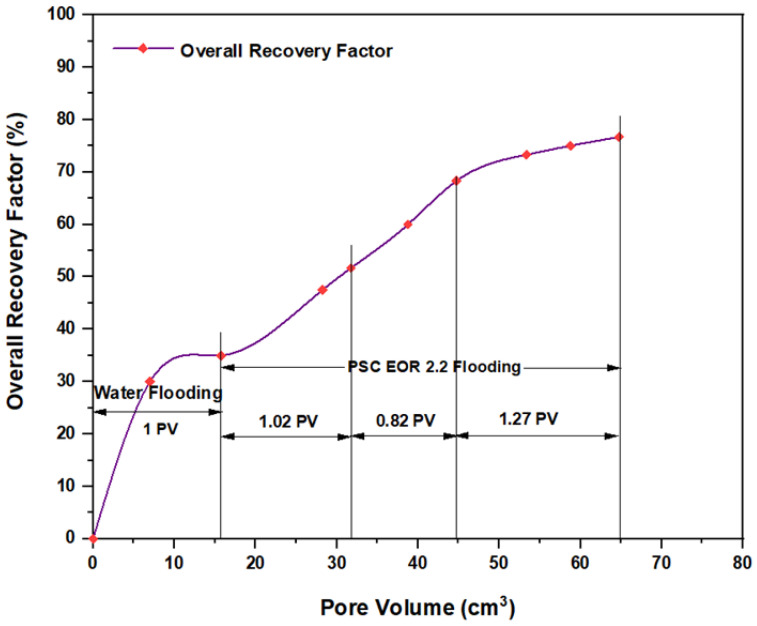
Oil extracted after water and surfactant flooding at 60 °C. The surfactant solution consisted of PSC EOR 2.2 at 0.98 wt.%.

For the Mits-5L001 surfactant (0.01 wt.%), the RF was slightly above 1.2 (1.239) and the RFF was below 1.2 (1.0784), but the adsorption rate was less than 0.4; therefore, this surfactant was considered satisfactory in adsorption and was used in the final ASP slugs. For PSC^TM^ HOMF (0.01 wt.%), the RF, RFF, and adsorption rates indicated its successful application. PSC EOR 2.2 (0.01 wt.%) also showed good performance, as the three mentioned criteria for adsorption were satisfied. For Dekasurf SF 9136 (0.01 wt.%), both the RF and RFF values were satisfactory (<1.2), but the adsorption rate was above the recommended value. Despite fulfilling two criteria out of three, this surfactant was excluded from the final ASP tests. This was because this product appeared to be more affected by the temperature, as demonstrated by the compatibility test. In this test, its quantity was reduced during the fourth and fifth days due to the continued exposure to heating at 60 °C. Moreover, this was confirmed in the direct injection test for this surfactant, as shown in Figure 21. Results demonstrated that the oil recovery portion was the smallest “in value” in comparison to the other surfactants involved in flooding, as shown in Figure 22, Figure 23 and Figure 24.

### 3.5. ASP Flooding Using Indonesian Surfactants

**Figure 25:**
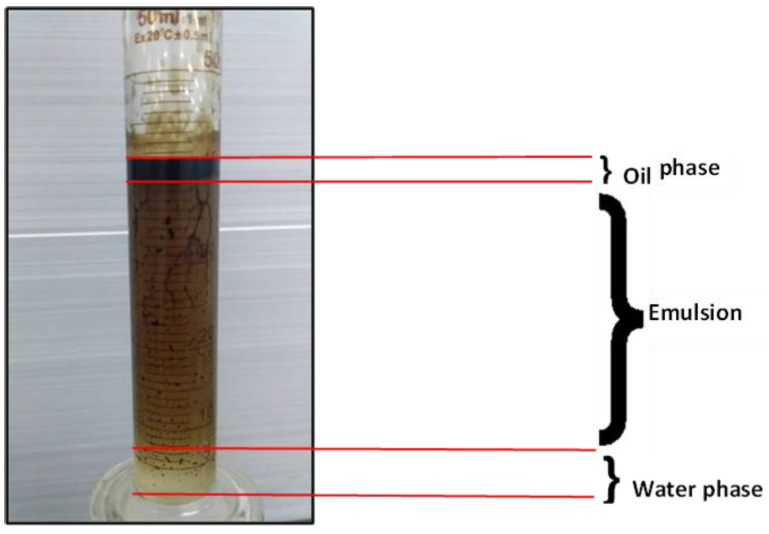
Oil extracted after water and polymer flooding at 60 °C. The polymer solution consisted of 2000 ppm HPAM and 0.6 wt. % PYNPs.

It is evident from the results that polymer flooding with HPAM and PYNPs/CSNPs gave the best results in terms of oil recovery in comparison to the other components. Despite the fact that the difference in oil recovery between these nano-polymers (PYNPs and CSNPs) was not large, the combination that contained PYNPs was more stable and led to higher oil recovery. This is because Windsor type 3 was achieved, as seen in Figure 25. The presence of this pattern indicates more oil recovery and the best applied recovery operation [17].

In order to improve the performance of Dekasurf SF 9136 manufactured in Riau, Indonesia, which was previously eliminated from the ASP experiments according to the adsorption and compatibility tests, surfactant flooding was performed with this product at 60 °C. As shown in Figure 21, the amount of oil recovered after water flooding was small (only 10.84 wt.%), and this quantity was not considered economically significant. This confirmed that this component was not suitable for adoption in the final ASP formation.

At the beginning of the flooding operations, water flooding was initiated until one pore volume (PV) of water (salinity 100 ppm) had been injected. Then, ASP flooding with different combinations of nano-polymers, alkaline, and special surfactants (that were obtained from PT SPR Langgak Company located in Riau, Indonesia) was started until 2 PV of chemical solution was injected for each cycle. The optimum concentrations for PYNPs and CSNPs with 2000 ppm HPAM were found, according to a study performed by Al-Jaber et al. [2], to be 0.6 wt.% and 0.8 wt.%, respectively. Based on the aforementioned study, the ideal concentrations for the remaining elements (alkaline and surfactants) were identified using critical micelle concentration (CMC) and IFT measurements with standard paraffin oil. The results of these measurements are illustrated in Table 4.

**Figure 26:**
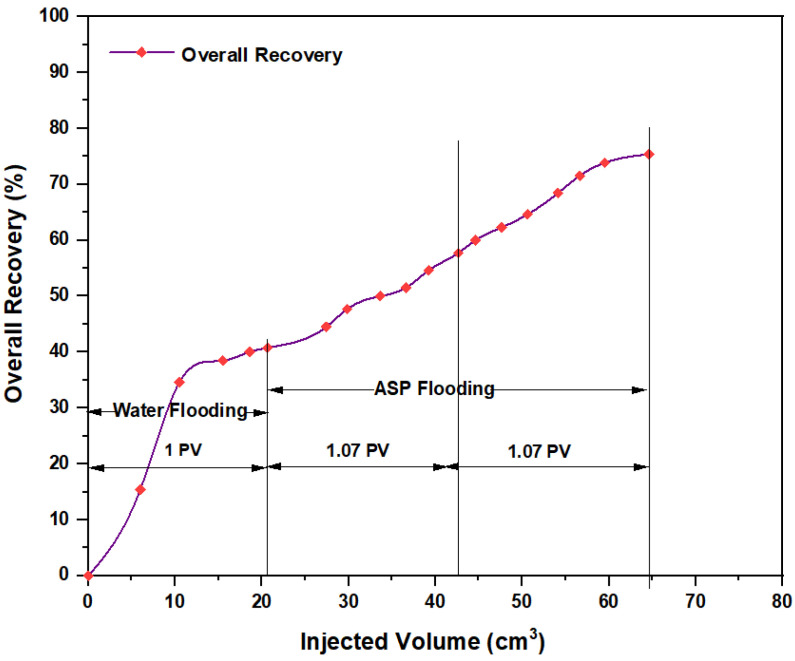
Overall oil recovery versus injected volume for NaOH (1.28 wt.%)–PSC HOMF (0.63 wt.%)–HPAM (2000 ppm) plus PYNPs (0.6 wt.%) at 60 °C after water flooding.

**Figure 27:**
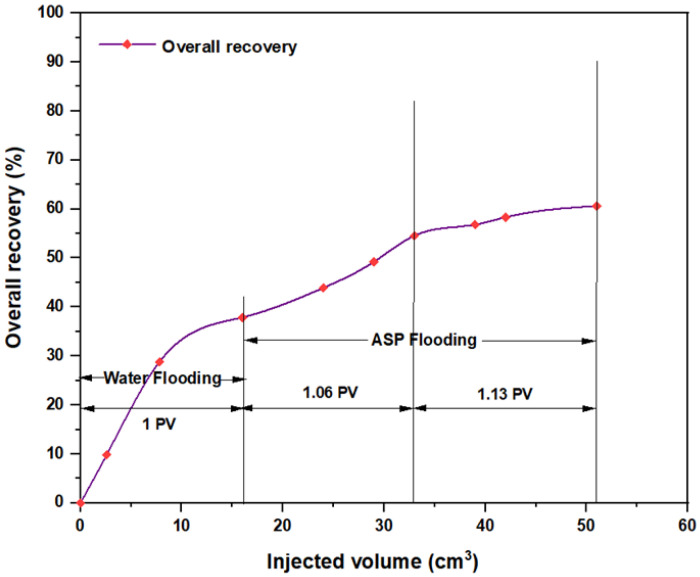
Overall oil recovery versus injected volume for NaOH (1.28 wt.%)–Mits-5L001 (1.0 wt.%)–HPAM (2000 ppm) plus PYNPs (0.6 wt.%) at 60 °C after water flooding.

The first tested combination for ASP flooding consisted of NaOH (1.28 wt.%) as the alkaline, PSC HOMF (0.63 wt.%) as the surfactant, and 2000 ppm HPAM with 0.6 wt.% as a hybrid polymer. As seen in Figure 26, the overall oil recovery for water flooding was 40.77%. After implementing ASP flooding with the above slug, the oil recovery increased randomly as the injected volume increased until it reached a maximum value of 75.38% when 2PV was injected into the core. Therefore, the net incremental improvement in oil recovery after water flooding was 34.61%. In other words, ASP flooding with the aforementioned combination was successful in recovering more oil than that obtained by water flooding. The other inspected slug was composed of NaOH (1.28 wt.%) as the alkaline, Mits-5L001 (1.0 wt.%) as the surfactant, and 2000 ppm HPAM with 0.6 wt.% as a hybrid polymer. As shown in Figure 27, the oil recovered from the injection of 1 PV of water (salinity 100 ppm) was 37.88%. The overall recovery after implementing 2 PV of ASP flooding was 60.61%, so the incremental oil recovery for this slug was 22.73%, which was lower than that obtained from water flooding.

**Figure 28:**
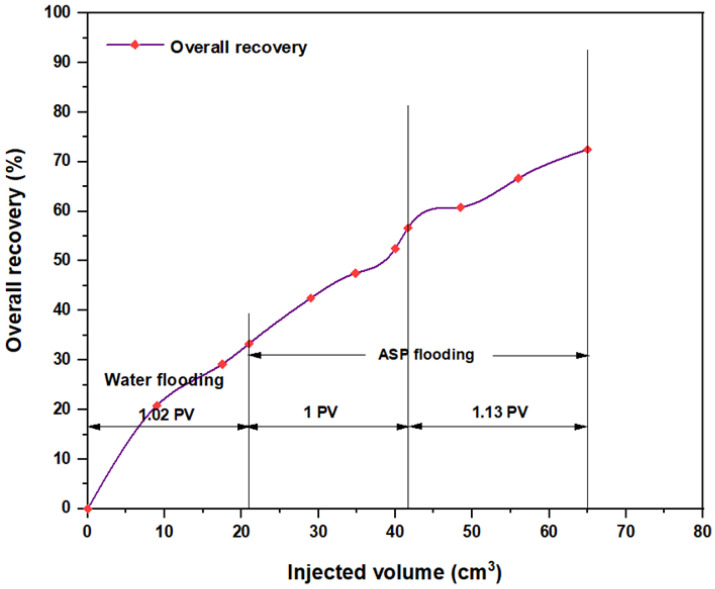
Overall oil recovery versus injected volume for NaOH (1.28 wt.%)–PSC EOR 2.2 (0.98 wt.%)–HPAM (2000 ppm) plus PYNPs (0.6 wt.%) at 60 °C after water flooding.

For the ASP combination that consisted of NaOH (1.28 wt.%) as the alkaline, PSC EOR 2.2 (0.98 wt.%) as the surfactant, and 2000 ppm HPAM with 0.6 wt.% PYNPs as the hybrid polymer, as shown in Figure 28, the overall water flooding recovery was 33.33%. After implementing ASP flooding, the overall oil recovery reached 72.5% after the injection of 2 PV of ASP chemicals. This recovery was the highest in comparison to all other combinations, as seen from Table 5. Thus, this slug was considered the best for the recovery of significant quantities of oil. The net incremental recovery for this combination was 39.17% after subtracting the ratio obtained by water flooding.

**Figure 29:**
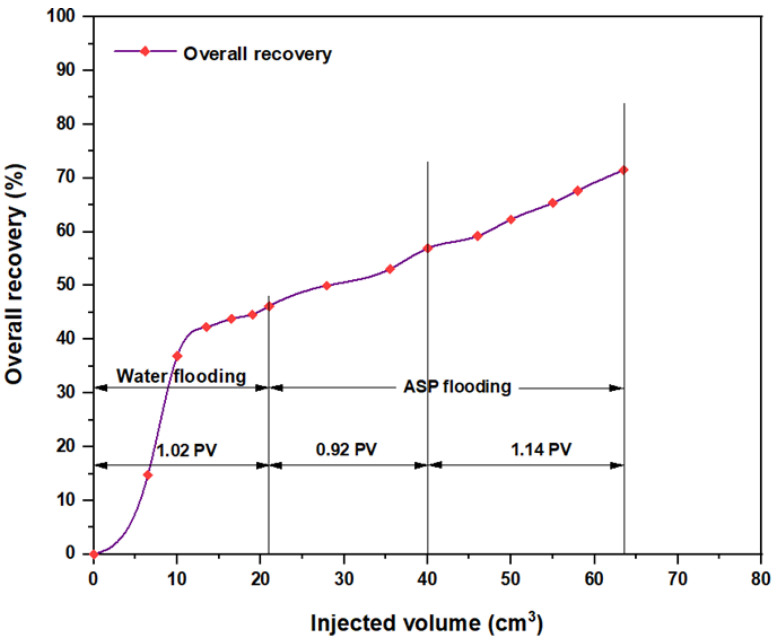
Overall oil recovery versus injected volume for Na_2_CO_3_ (0.9 wt.%)–HOMF (0.63 wt.%)–HPAM (2000 ppm) plus PYNPs (0.6 wt.%) at 60 °C after water flooding.

**Figure 30:**
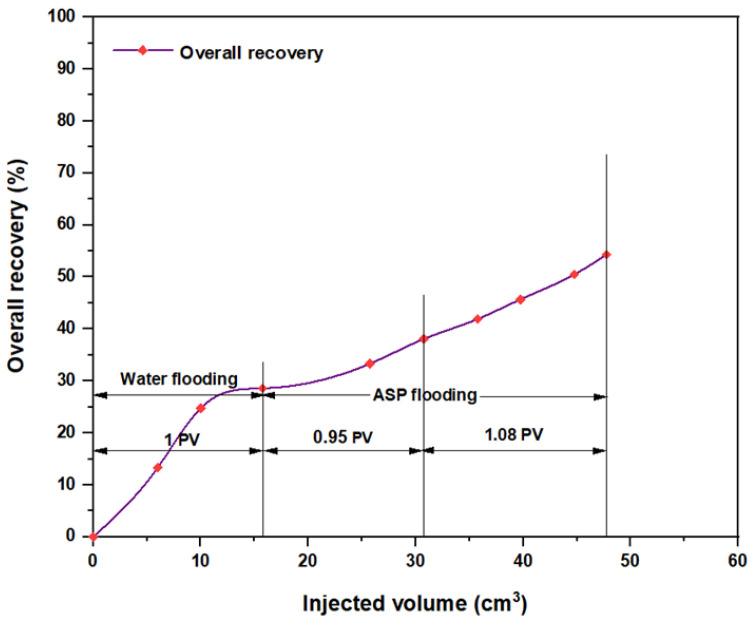
Overall oil recovery versus injected volume for Na_2_CO_3_ (0.9 wt.%)–Mits-5L001 (1.0 wt.%)–HPAM (2000 ppm) plus PYNPs (0.6 wt.%) at 60 °C after water flooding.

For the combination that consisted of weak alkali Na_2_CO_3_ (0.9 wt.%), alongside the other components (PSC HOMF and hybrid polymer), the oil recovery after implementing brine flooding was 46.15%. After ASP flooding, the overall recovery increased to 71.54%. The net incremental recovery, therefore, for this combination was 25.39%, as shown in Figure 29. The other combination that included Mits-5L001 (1.0 wt.%) as well as the weak alkali and the hybrid polymer is shown in Figure 30. The water flooding recovery for 1 PV brine injection was 28.57%, whereas the ASP recovery after implementing 2 PV was 54.29%. From this study, it is obvious that oil recovery using a weak alkali is less successful than that using a strong alkali such as NaOH, and this corresponds with a number of previous studies [25,26,27,28,29]. The incremental recovery for this combination was 25.72%.

**Figure 31:**
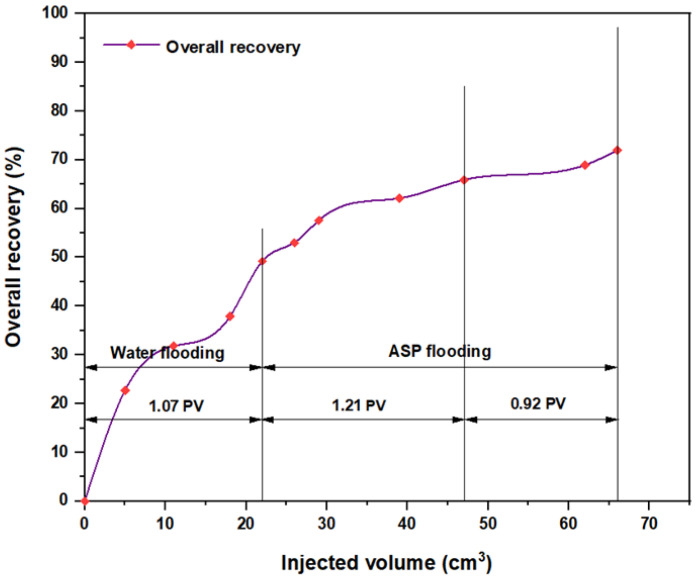
Overall oil recovery versus injected volume for Na_2_CO_3_ (0.9 wt.%)–PSC EOR 2.2 (0.98 wt.%)–HPAM (2000 ppm) plus PYNPs (0.6 wt.%) at 60 °C after water flooding.

The oil recovery for the ASP formulation that used PSC EOR 2.2 (0.98 wt.%) as a surfactant, alongside the weak alkali and hybrid polymer, is shown in Figure 31. The overall water flooding recovery was 49.24%, and that for ASP injection was 71.97%, which is a high recovery ratio. A possible explanation for this high recovery is that the PSC EOR 2.2 surfactant improved chemically the properties of the Na_2_CO_3_ alkali, which enhanced the oil recovery. Another possible explanation is related to the fact that the mixing of this surfactant with the Na_2_CO_3_ alkali increased the microscopic sweep efficiency and altered the wettability of the used core sample from oil-wet to water-wet [30,31]. A third possible reason is that the use of this weak alkali in the ASP combination together with this surfactant led to the formation of a so-called “in-situ” surfactant through the reaction of the alkaline agent with the acidic components available in the crude oil, and this activated a new series of mechanisms that improved the oil recovery as a result [32,33,34]. The net incremental oil recovery for this ASP formula was 22.73%.

**Figure 32:**
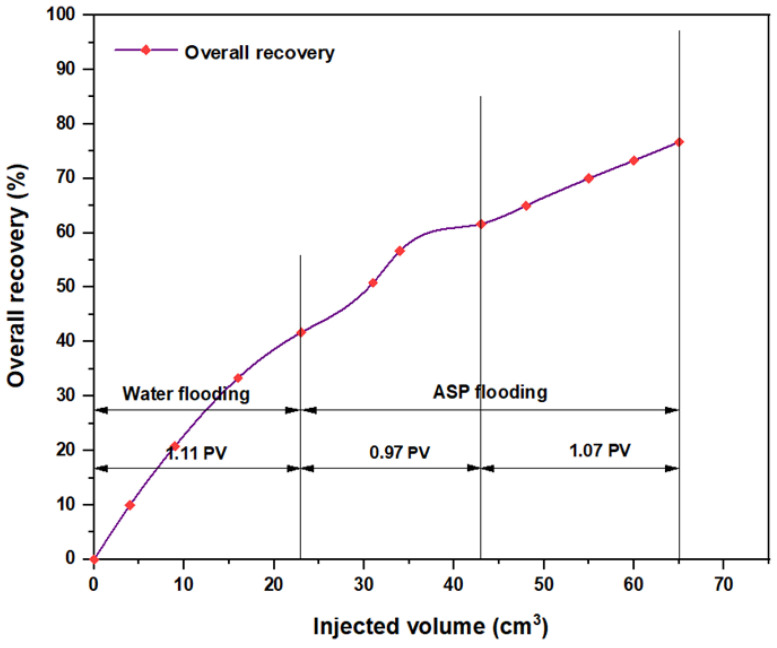
Overall oil recovery versus injected volume for NaOH (1.28 wt.%)–PSC HOMF (0.63 wt.%)–HPAM (2000 ppm) plus CSNPs (0.8 wt.%) at 60 °C after water flooding.

**Figure 33:**
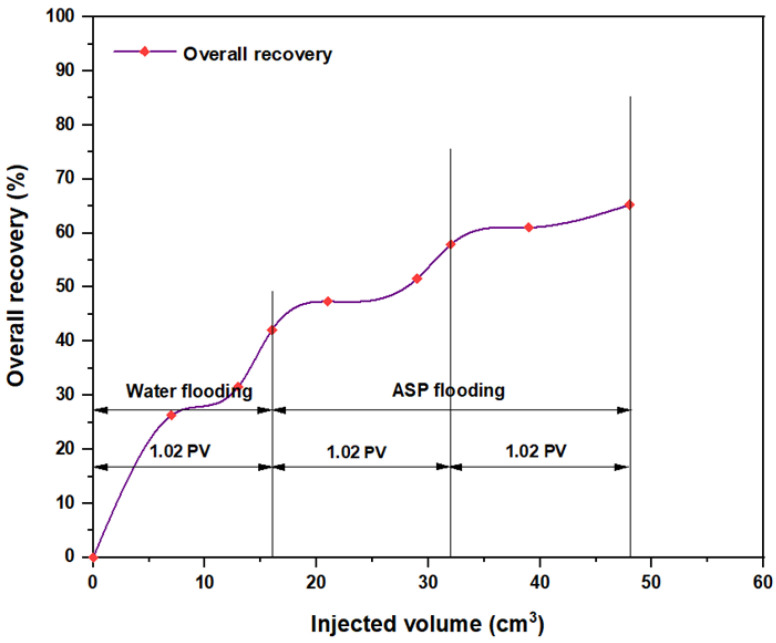
Overall oil recovery versus injected volume for NaOH (1.28 wt.%)–Mits-5L001 (1.0 wt.%)–HPAM (2000 ppm) plus CSNPs (0.8 wt.%) at 60 °C after water flooding.

**Figure 34:**
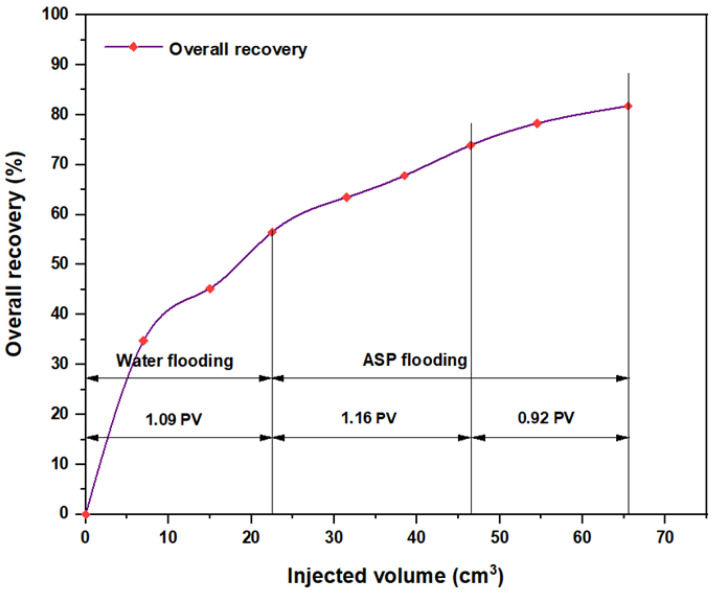
Overall oil recovery versus injected volume for NaOH (1.28 wt.%)–PSC EOR 2.2 (0.98 wt.%)–HPAM (2000 ppm) plus CSNPs (0.8 wt.%) at 60 °C after water flooding.

**Figure 35:**
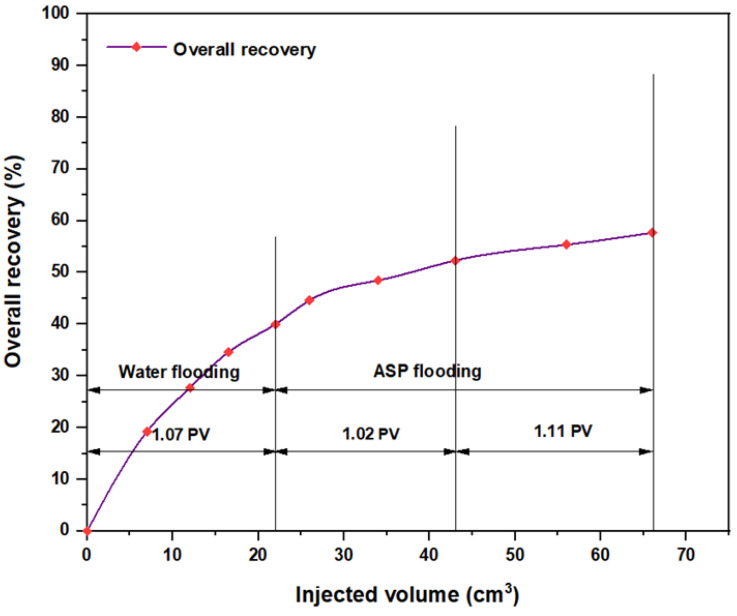
Overall oil recovery versus injected volume for Na_2_CO_3_ (0.9 wt.%)–HOMF (0.63 wt.%)–HPAM (2000 ppm) plus CSNPs (0.8 wt.%) at 60 °C after water flooding.

**Figure 36:**
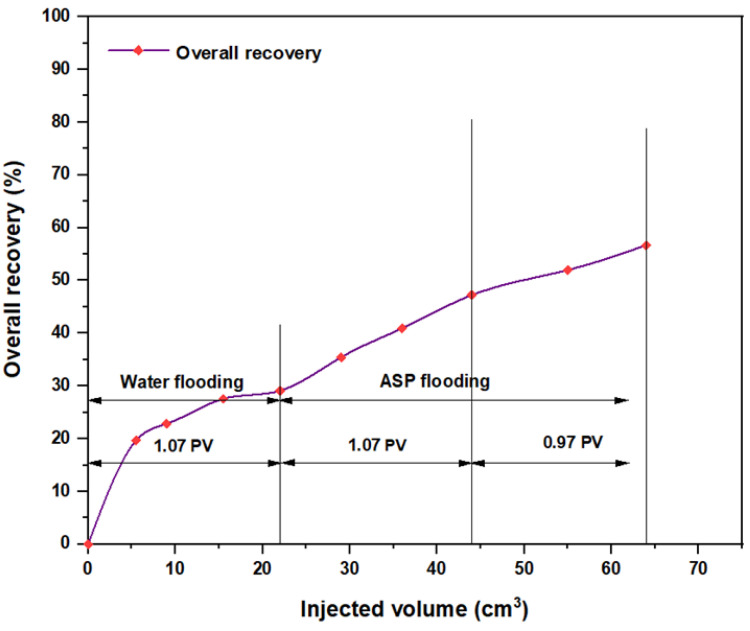
Overall oil recovery versus injected volume for Na_2_CO_3_ (0.9 wt.%)–Mits-5L001 (1.0 wt.%)–HPAM (2000 ppm) plus CSNPs (0.8 wt.%) at 60 °C after water flooding.

**Figure 37:**
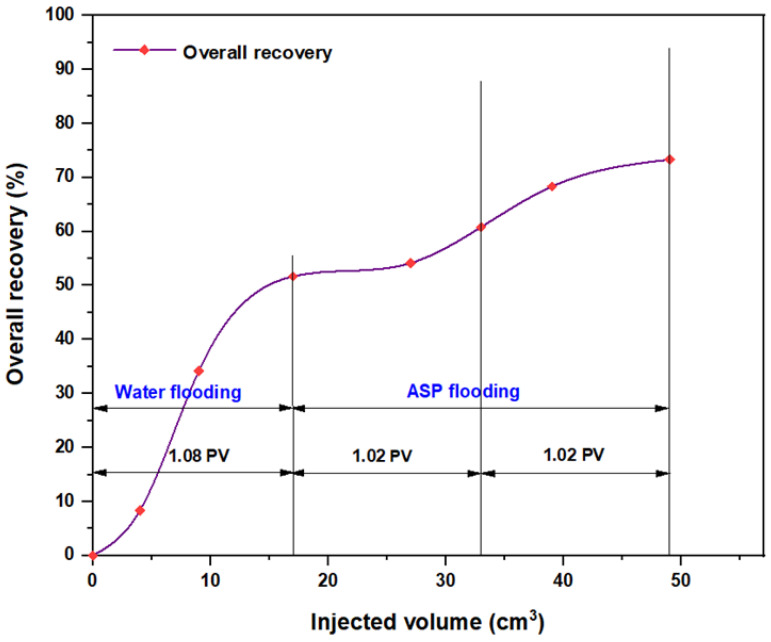
Overall oil recovery versus injected volume for Na_2_CO_3_ (0.9 wt.%)–PSC EOR 2.2 (0.98 wt.%)–HPAM (2000 ppm) plus CSNPs (0.8 wt.%) at 60 °C after water flooding.

The same slugs, components, and concentrations were used with the hybrid polymer, which consisted of 2000 ppm HPAM with CSNPs at a concentration of 0.8 wt.%, and the relationships among the overall oil recovery and the injected volume for the same components are shown in Figure 32, Figure 33, Figure 34, Figure 35, Figure 36 and Figure 37. Generally, the recovery percentage using this nano-polymer combination was somewhat lower than that with the first nano-polymer that used PYNPs, which indicated that the first combination was more efficient in extracting a significant quantity of oil. A possible reason for this is that the PYNP particles created a more stable diffusion force in comparison to CSNP particles due to their large surface area [35]. A second possible reason is that the PYNPs together with HPAM had more potential than CSNPs with HPAM in reducing the oil viscosity and improving the mobility ratio, and they were also more effective in altering the rock wettability towards water-wet conditions [36,37]. In addition to the higher recovery obtained using these two nanoparticle solutions with HPAM, the operational costs related to ASP flooding can be reduced, since these types of nanoparticles are abundantly available in nature, especially in this region of Asia. Therefore, when applied in large-scale operations, oil recovery may be significantly increased if the conditions of the oilfield are the same as those tested in this study.

Table 5 shows a summary of the incremental oil recovery after water flooding and ASP flooding for all slugs and chemicals used in this study. According to a study performed by Negin C. et al. [38], the types of nanoparticles used in this study are considered “organic particles”, which are different from inorganic particles and metal oxide particles; therefore, the latter two types of nanoparticles were not considered in this study. As seen in the table, there were two combinations of ASP that achieved higher recovery. The first was the combination that contained PYNPs, which consisted of NaOH (1.28 wt.%), PSC EOR 2.2 (0.98 wt.%), and 2000 ppm HPAM with 0.6 wt.% PYNPs. This combination achieved incremental oil recovery of 39.17% after water flooding, which was the highest in this study. The second combination was the one that contained CSNPs, which consisted of NaOH (1.28 wt.%), PSC HOMF (0.63 wt.%), and 2000 ppm HPAM with 0.80 wt.% CSNPs. An incremental oil recovery rate of 35% was obtained with this slug. Therefore, these two ASP combinations are considered the best ones that can increase oil recovery when implemented in ASP operations at a temperature of 60 °C. In addition, the flooding process using these types of seeded starch nanoparticles in polymer formations consisting of HPAM is regarded as an “economical process” as the tubers for cassava and purple yam are affordable and abundant.

Various factors contributed to the enhancement in oil recovery achieved by these two ASP combinations. One significant factor was the reduction in interfacial tension between the oil and water phases. The two surfactants PSC EOR 2.2 and PSC HOMF, obtained from the PT SPR Langgak Oil Company, played a crucial role in achieving this reduction. Another contributing factor was the successful alteration of the wettability, as evidenced by the decreased contact angle observed in the wettability test. For PSC EOR 2.2, the contact angle was reduced to 26.2°, while, for PSC HOMF, it was reduced to 37.2°. Furthermore, effective mobility control was achieved through the combination of HPAM with PYNPs/CSNPs, resulting in the improved viscosity of the injected water in these two combinations. Additionally, the PYNP-containing combination demonstrated the generation of an oil-in-water emulsion, which enhanced the oil recovery by increasing the contact area between the oil and displacing fluid, as illustrated in Figure 21. Moreover, the alkaline agent (NaOH) used in the two ASP formulations reacted with the acidic components available in the crude oil, such as naphthenic acids. These reactions led to the formation of an in-situ surfactant, a soap-like compound that mixed with the original surfactant present in the injection stream. This further enhanced the solubility of the oil, contributing to improved recovery.

## 4. Materials and Methods

### 4.1. Materials

#### 4.1.1. Buff Berea Core Samples

Atama Tech Sdn. Bhd. supplied five Buff Berea core samples. Furthermore, the reservoir laboratory provided two additional cores. These core samples were used in experiments that involved water and ASP flooding. They had comparable features to the original sandstone in the Langgak oilfield in Sumatra, Indonesia. The core sample’s length was 3 inches, and its diameter was 1.5 inches. The properties are shown in Table 6.

#### 4.1.2. Crude Oil

Crude oil with an °API of 31.9 was obtained from the Langgak oilfield in Sumatra, Indonesia. The viscosity of the crude oil was approximately 43.668 cp, and the oil phase was solid at typical temperatures (25 °C). Most Indonesian crude oils have pour points and wax content that range between 35 °C and 40 °C and 20–25%, respectively. Such oil necessitates the use of specialized technologies in order to keep it in a liquid state. To achieve this, this oil was treated with a chemical solution called Fsol at a 1:1 ratio. This chemical solution was purchased from Innochems Technologies Sdn. Bhd. in Johor, Malaysia.

Fsol has the capacity to lower the viscosity of oil and convert it into a liquid at room temperature without affecting its primary qualities. This may be accomplished by combining the crude oil in the ratio stated previously with this solution and then stirring the treated oil for approximately 10 min with a magnetic stirrer. To guarantee that the modified oil had a homogeneous composition after being combined with Fsol, it was heated in an oven at 60 °C for roughly 30 min before being used in ASP flooding.

#### 4.1.3. Partially Hydrolyzed Polyacrylamide (HPAM)

HPAM is the most commonly used polymer in EOR processes because of its relative fixed viscosity at low to moderate temperatures (around 693 to 787 mPa.s at 35 °C) and its stable chemical and physical properties. The synthesized HPAM used in this study had a molecular weight of 27.7 × 106 g/mol, which is considered suitable for EOR applications and further for ASP flooding. HPAM with a concentration of 0.5% in aqueous solution (brand R&M) was purchased from Tricell Bioscience Resources Co., Skudai, Johor, Malaysia.

#### 4.1.4. Acetic Acid (CH_3_COOH)

Glacial acetic acid (CH_3_COOH) with purity of 99% (*w*/*w*) was supplied by QREC (Asia) Sdn. Bhd., Selangor, Malaysia. Acetic acid was used in the manufacturing of purple yam and cassava nanoparticles. The independent parameters that can affect the production of starch nanoparticles are the acid concentration (mol/L), temperature (°C), and time of operation (days). The accessibility range for these parameters was classified according to the results obtained from previous studies [39,40,41], as shown in Table 7.

#### 4.1.5. Sodium Hydroxide (NaOH) and Sodium Carbonate (Na_2_CO_3_)

In the current study, two different alkalis were tested: NaOH and Na_2_CO_3_. NaOH was provided by ASIA (QREC) Sdn. Bhd., Selangor, Malaysia, whereas Na_2_CO_3_ was purchased from ASIA CHEMIE (QREC) Co., Ltd., Chonburi, Thailand. The properties of sodium hydroxide and sodium carbonate are illustrated in Table 8.

#### 4.1.6. Sodium Dodecyl Sulphate (SDS) and Sodium Dodecyl Benzene Sulfonate (SDBS)

SDS is the most inspected material in oil recovery as an anionic surfactant. It can be obtained as a powder or pellet and can be used in biotechnology and biochemistry. SDS was provided by ASIA CHEMIE (QREC) Co. Ltd., Thailand. In addition, SDBS with the molecular formula C_18_H_29_NaO_3_S was used. SDBS is a yellow, oily component with micro-toxicity. SDBS is considered neutral, more sensitive to water hardness, and an anionic surfactant. SDBS with a molecular weight of 348.48 g/mol was purchased from Central Drug House (P) Ltd., New Delhi, India.

#### 4.1.7. Surfactants Obtained from PT SPR Langgak Company in Indonesia

Four collections of surfactants at certain concentrations were obtained from the PT SPR Langgak company in Sumatra, Riau, Indonesia. In this research, not all supplied surfactants were processed for the final flooding, as some of them did not show improved performance. The supplied surfactant combinations were named Mits-5L001, PSC HOMF, Dekasurf SF 9136, and PSC EOR 2.2.

#### 4.1.8. Purple Yam Tubers

Eighteen kilograms of purple yam tubers were purchased from a domestic Johor market. It is scientifically known as *Dioscorea alata* and is also known as greater yam or water yam. It is one of several yam species that have been domesticated and farmed for their starchy tubers throughout Southeast Asia and New Guinea. The average particle size of the PYNPs that were used in the flooding experiments was 363.12 nm and the concentration was 0.6 wt.% (*w*/*w*) [2].

#### 4.1.9. Native Cassava Starch

One kilogram of native cassava starch was obtained from the domestic market. Cassava is a versatile vegetable that is extensively consumed across the world, and it is also the source of tapioca starch. Cassava starch is a white powder formed from dehydrated and dried tapioca after it has been removed. The average particle size of the CSNPs was 52.92 nm, and the concentration that was used in the flooding experiments was 0.8 wt.% (*w*/*w*) [2].

### 4.2. Methods

#### 4.2.1. Determining the Wettability of Core Samples

In order to test the wettability of the surfactants, alkalis, and nano-polymers using two types of nanoparticles synthesized according to a method described by Al-Jaber et al. [2], Buff Berea core samples were used. The crude oil was supplied by the Langgak oilfield, and it was mixed with Fsol (1:1) to reduce its viscosity and ensure that it remained in a liquid state at ambient temperature. In order to ensure the homogeneous composition of this diluted oil, it was stirred for around 10 min using a magnetic stirrer and then placed in an oven at 60 °C for around 30 min before being used.

The core samples were soaked completely in oil for approximately three days. This was to ensure full or complete saturation with crude oil. At this point, the wettability of the core samples was measured with an optical contact angle instrument. The measured contact angle was found to be higher than 90° as the core samples were fully saturated with crude oil. After this, the alkali, surfactant, and nano-polymer solutions were prepared at different concentrations from these components. These concentrations were based on the CMC technique, which aimed to ensure lower interfacial tension between these components and the standard paraffin oil. Then, the same core samples that were saturated with crude oil were soaked in the prepared solutions for around three days until they became saturated. To evaluate the wettability, the core samples were immersed in the prepared solutions and the contact angle was calculated using an optical instrument, and its value was less than 90°, which was considered suitable for the application of the alkalis, surfactants, and nano-polymers in ASP flooding, especially as this contact angle was reduced further as the wettability changed from oil-wet to water-wet. To perform this activity, one core sample was cut into six small discs, each with the same diameter (1.5 inches) and a length of 0.5 inches. These discs were labeled numerically (1, 2, 3, etc.), and each one was placed in direct contact (through soaking) with a specific solution.

#### 4.2.2. Compatibility for Surfactants

**Figure 38:**
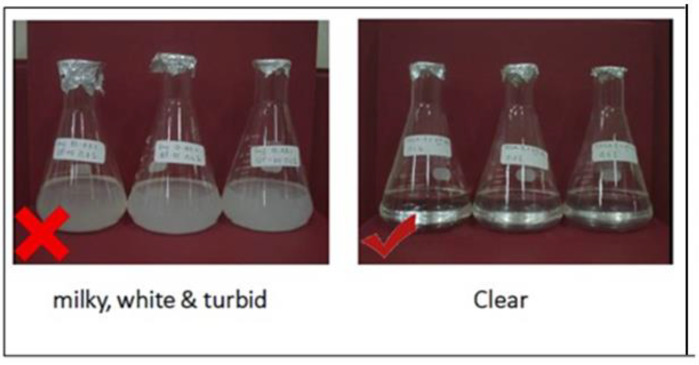
Compatibility test for surfactant combinations.

In this method, the tested surfactants were assessed with the formation/injection water to ensure that they were compatible with it to a certain degree. This was achieved by preparing a solution of surfactant at a certain concentration (mostly 1 wt.%) in a 1000 mL beaker. The prepared solutions were kept at the reservoir temperature of the Langgak oilfield (60 °C) by placing them inside a standard oven. In this test, the change in solubility of the surfactant solutions was observed for five consecutive days. In this test, a satisfactory result was a clear solution in a single phase with no sedimentation, as shown in Figure 38.

#### 4.2.3. Thermal Stability

In this test, the performance of the surfactant solutions was evaluated at the reservoir temperature (60 °C). The viscosity of the solutions was measured using Brookfield RST rheometer manufactured in Middleboro, Massachusetts, United States. The SFT of the solutions was estimated using a KRUSS EasyDyne tensiometer K20 by implemeting Wilhelmy plate method. The Brookfield RST rheometer utilizes a rotational measurement method to determine the viscosity. It employs a spindle that rotates within a sample, measuring the torque required for rotation. The torque measurement is then used to calculate the viscosity of the sample based on the rheological properties and behavior of the material being tested, which involves measuring the force required to vertically lift or immerse a plate or wire in a liquid. This method is utilized to accurately assess the interfacial tension and surface tension properties of different liquids. In order to obtain consistent and stable values for the viscosity and SFT at the reservoir temperature, these values were recorded and had to remain unchanged for a total period of 10 days. This was achieved by placing the solutions in the oven at 60 °C for 10 days and measuring the values of viscosity and SFT each day. The concentrations of surfactants varied from 0.4 to 1.0 wt.% in this test.

#### 4.2.4. Adsorption and Injectivity Test

**Figure 39:**
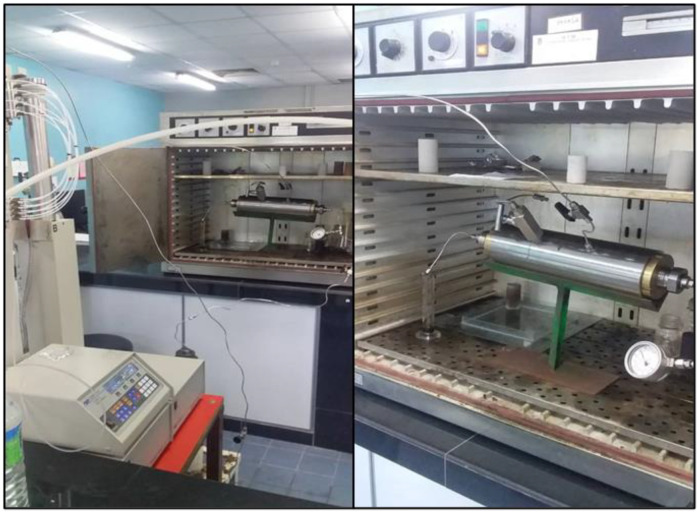
Main core-flooding apparatus used in experimental work.

In this test, the interactions between reservoir rocks engaged by Buff Berea core samples and injected chemical solutions were evaluated. The instruments used for this test were a core-flooding unit (Figure 39) and a UV–VIS spectrophotometer. The dimensions of the inserted core sample had to be known. The inspected core was inserted into a desiccator, vacuumed, and saturated with brine water (100 ppm). After this, injection was initiated with the brine water followed by the surfactant (or polymer) solutions at a volumetric flow rate (Q) equal to 3.5 cc/min. The injection process was continued until the pressure drop between the inlet and outlet became constant. The injection effluent was collected in a graduated cylinder, and the contents of this effluent were evaluated for every 5 mL volume. After measuring the necessary concentrations, the value of the dynamic adsorption value for each core sample was estimated.

In order to calculate the injectivity, the differential pressure (ΔP) between the inlet and outlet was recorded. Then, the value of RF was calculated for the polymer (or surfactant) solutions. After this, a re-injection operation was performed in the inspected core sample with brine water (salinity 100 ppm) in order to calculate the RRF. To assess the rheological properties of the tested polymers (or surfactants) through the core samples, the differential pressure between the inlet and outlet (ΔP) was measured until it became constant. Herein, the RF and RRF were determined using the following equations [42]:

RF = ΔP_Polymer_/ΔP_Brine_ (1)

RFF = ΔP_Brine after polymer injection_/ΔP_Brine_ (2)

where ΔP_Brine_ is the differential pressure of brine before polymer (or surfactant) injection, ΔP_polymer_ is the differential pressure for polymer (or surfactant) injection, and ΔP_Brine_ after polymer injection represents the ΔP of resumed brine injection after polymer (or surfactant) injection. For successful performance, the following conditions had to be met [43]:

I. Adsorption value < 400 μg/g;
II. RF and RRF < 1.2.

#### 4.2.5. Water and ASP Flooding

Flooding experiments were initiated for the final selected combinations of ASP, and the nano-polymer solution consisted of HPAM and PYNPs or CSNPs. The first nano-polymer solution consisted of 2000 ppm HPAM plus 0.60 wt.% PYNPs. The second one was composed of 2000 ppm HPAM and 0.80 wt.% CSNPs. The Buff Berea core samples were vacuumed and located inside the saturation vessel. The vacuum operation was performed for around three hours using a vacuum pump to remove air from inside the core samples. Brine with salinity of 100 ppm was produced by dissolving 10 g of NaCl in 1000 mL of distilled water. The vacuum pump was used again to introduce the brine into the saturation vessel.

When the brine solution had completely filled the open space inside the saturation vessel, the surplus brine began to discharge and collect in a conical flask. The vacuum pump was then turned off, and the saturation vessel was attached to a Teledyne pump. Following the operation of the Teledyne pump, the pressure within the saturation vessel was progressively increased by injecting 8 cm^3^/min of water. When the pressure inside the vessel approached approximately 2200 psi, the accumulator’s brine injection was stopped. The saturation vessel was kept at this pressure for 2–3 days to ensure that the core samples were completely saturated with the brine.

After the saturation of the core samples with brine, they were processed for crude oil, where crude oil mixed with Fsol was injected. Nitrogen gas was pumped into the confining vessel to assist in spreading the oil inside the sandstone pores. The Teledyne pump was used for the injection of oil at a volumetric flow rate of 7–10 cm^3^ per minute. The injection output was collected in a 50 mL collecting cylinder. The amount of water resulting from oil injection is equal to the amount of OOIP. This is because of the law of conservation of mass: mass in is equal to mass out. After the core sample was saturated with crude oil, water flooding (brine) was started by injecting 5–8 cm^3^/min of brine into the core sample. Every three minutes, the output from water flooding was checked in the output cylinder, and its quantity was measured. Water flooding continued until one PV of water was injected.

After this, ASP flooding was initiated, which was related to the injection of HPAM-nano-starch polymers together with alkaline and surfactants. The volumetric flow rate for ASP flooding was 3–3.5 cm^3^/minute, and the output of flooding was examined every three minutes as before. In order to maintain the temperature for water and ASP flooding around 60 °C, the confining vessel that contained the core sample was placed inside an electrical oven at 60 °C to simulate the temperature of the Langgak oilfield. The ASP flooding was allowed to continue until 2 PV was injected inside the core sample. The oil recovery percentage for this operation (Rf) was calculated using the following equation [24]:

Rf = (total volume of oil produced at collecting cylinder/OOIP) × 100%(3)

## 5. Conclusions

Notably, current field applications of ASP flooding have achieved incremental oil recovery of approximately 20–22% [43]. The objective of this study was to identify an optimal injection design pattern that could reduce costs while improving oil recovery. Various evaluation techniques were employed to assess the performance of different surfactants, and those showing favorable results were incorporated into the final ASP formulation. The performance of the surfactants obtained from the PT SPR Langgak Oil Company in Indonesia, including Mits-5L001, PSC^TM^ HOMF, Dekasurf SF 9136, and PSC EOR 2.2, was evaluated prior to their inclusion in the ASP experiments. Additionally, SDS and SDBS surfactants were evaluated but yielded unfavorable outcomes in rheology and adsorption tests. A unique type of nano-polymer was developed using cassava and purple yam starches, which was then combined with conventional HPAM at a concentration of 2000 ppm. The performance of the nano-polymer, comprising PYNPs at a concentration of 0.6 wt.% and CSNPs at a concentration of 0.8 wt.%, was found to be favorable. The final ASP combinations involved two types of alkali: NaOH (1.28 wt.%) as a strong alkali and Na_2_CO_3_ (0.9 wt.%) as a weak alkali. The combination with the strong alkali yielded superior results in terms of achieving higher recovery.

Evaluation methods for the surfactants encompassed compatibility and wettability alterations for Buff Berea core samples, thermal stability, and adsorption with injectivity tests. The Dekasurf SF 9136 surfactant exhibited poor performance in compatibility, adsorption, and injectivity tests, with an adsorption rate exceeding 0.4 mg/g, leading to its exclusion from the final ASP flooding experiments. Similarly, SDS and SDBS showed poor performance in the rheology tests and were also excluded. After conducting a series of ASP flooding experiments using the remaining surfactants, it was determined that the combination of NaOH (1.28 wt.%), PSC EOR 2.2 (0.98 wt.%), and the nano-polymer HPAM (0.2 wt.%) with PYNPs (0.6 wt.%) achieved the highest oil recovery among all other combinations, with a net incremental oil recovery of 39.17% after water flooding. Additionally, the combination of NaOH (1.28 wt.%), PSC HOMF (0.63 wt.%), and the nano-polymer HPAM (0.2 wt.%) with CSNPs (0.8 wt.%) yielded the second-highest recovery, with an incremental recovery of 35% after waterflooding. This indicates that the nano-polymer consisting of PYNPs and HPAM was more effective in extracting oil from core samples compared to the second combination. Therefore, the procedure outlined in this research has the potential to lead to greater levels of oil recovery and significantly improve oil extraction while reducing operational costs.

## Figures and Tables

**Table 1:** Contact angle for 6 discs of Buff Berea core sample submerged in crude oil at 60 °C.

Disc Label	Left Contact Angle (deg.)	Right Contact Angle (deg.)	Average Contact Angle (deg.)
Disc 1	71.7	70.7	71.2
Disc 2	76.7	73.7	75.2
Disc 3	80.4	79.2	79.8
Disc 4	85.0	80.4	82.7
Disc 5	99.3	100.4	99.85
Disc 6	83.2	84.9	84.05

**Table 2:** Contact angles for 6 discs of Buff Berea core sample submerged in crude oil and different surfactants/nano-polymers at 60 °C.

Disc Label	Solution Type	Left Contact Angle (deg.)	Right Contact Angle (deg.)	Average Contact Angle (deg.)
Disc 1	PSC HOMF (0.63 wt.%)	39.3	35.0	37.15
Disc 2	Dekasurf SF 9136 (1.24 wt.%)	33.8	39.8	36.8
Disc 3	Mits-5L001 (1.0 wt.%)	23.9	28.0	25.95
Disc 4	PSC EOR 2.2 (0.98 wt.%)	22.9	29.4	26.15
Disc 5	HPAM (2000 ppm) + PYNPs (1.25 wt.%)	48.0	46.6	47.3
Disc 6	HPAM (2000 ppm) + CSNPs (1.25 wt.%)	55.8	54.3	55.05

**Table 3:** Results for adsorption and injectivity of tested surfactants and polymers.

Chemicals Inspected	Material Injected	Pressure Difference, ΔP, atm	Volumetric Flow Rate, cm^3^/sec	Conc. wt.%	Permeability, mD	Resistance Factor, RF	Residual Resistance Factor, RFF	Adsorption Rate, mg/g
HPAM	HPAM	0.0851	0.005	0.10	109.25	0.4098 < 1.2 *	-	
	Brine Re-Injected	0.2674	0.0333	0.010	231.6	-	1.2885 > 1.2 **	-
HPAM + Purple Yam NPs	HPAM + Purple Yam NPs	0.1837	0.005	0.1 (HPAM) + 1.25 (PYNPs)	50.61	0.40298 < 1.2	-	
	Brine Re-Injected	0.54641	0.0333	0.01	113.32	-	1.1985 < 1.2	-
HPAM + Cassava NPs	HPAM + Cassava NPs	0.23816	0.005	0.1 (HPAM) + 1.25 (CSNPs)	39.04	0.35 < 1.2	-	
	Brine Re-Injected	0.56478	0.0333	0.01	109.63	-	0.83 < 1.2	-
SDS	SDS	0.1429	0.005	0.625	65.1	1.75 > 1.2	-	3.25 > 0.4 ***
	Brine Re-Injected	0.42529	0.0333	0.01	145.59	-	5.208 > 1.2	-
SDBS	SDBS	0.2688	0.005	0.5	34.59	1.681 > 1.2	-	3.0 > 0.4
	Brine Re-Injected	0.25177	0.0333	0.01	216.89	-	1.574 > 1.2	-
Mits-5L001	Mits-5L001	0.21502	0.005	0.10	43.24	1.239 > 1.2	-	0.3 < 0.4 ****
	Brine Re-Injected	0.18713	0.0333	0.010	330.89	-	1.0784 < 1.2	-
PSC HOMF	PSC HOMF	0.32322	0.005	0.10	28.764	0.475 < 1.2	-	0.28 < 0.4
	Brine Re-Injected	0.57158	0.0333	0.01	108.33	-	0.475 < 1.2	-
Dekasurf SF 9136	Dekasurf SF 9136	0.63963	0.005	0.10	14.54	0.752 < 1.2	-	0.5 > 0.4
	Brine Re-Injected	0.89820	0.0333	0.01	68.938	-	1.056 < 1.2	-
PSC EOR 2.2	PSC EOR 2.2	0.51034	0.005	0.10	18.218	0.42857 < 1.2	-	0.39 < 0.4
	Brine Re-Injected	1.29287	0.0333	0.01	47.893	-	1.0857 < 1.2	-

* <1.2 (favorable), ** >1.2 (unfavorable), *** >0.4 (unfavorable), **** <0.4 (favorable).

**Table 4:** Optimum concentrations for alkaline and surfactants [2].

S.	Component	Type of Component	Concentration (wt.%)
1	Sodium hydroxide (NaOH)	Alkaline	1.28
2	Sodium carbonate (Na_2_CO_3_)	Alkaline	0.90
3	PSC HOMF	Surfactant	0.63 wt.%
4	Dekasurf SF 9136	Surfactant	1.24 wt.%
5	Mits-5L001	Surfactant	1.0 wt.%
6	PSC EOR 2.2	Surfactant	0.98 wt.%

**Table 5:** Incremental oil recovery (%) for final ASP combinations tested in flooding experiments.

S.	Alkali	Surfactant	Polymer	Concentration of ASP Slug wt.%	Net Incremental Oil Recovery (%)
1	NaOH	PSC HOMF	(HPAM + PYNPs)	1.28 − 0.63 − (0.2 + 0.60)	34.61
2	NaOH	Mits-5L001	(HPAM + PYNPs)	1.28 − 1.0 − (0.2 + 0.60)	22.73
3	NaOH	PSC EOR 2.2	(HPAM + PYNPs)	1.28 − 0.98 − (0.2 + 0.60)	39.17
4	Na_2_CO_3_	PSC HOMF	(HPAM + PYNPs)	0.90 − 0.63 − (0.2 + 0.60)	25.39
5	Na_2_CO_3_	Mits-5L001	(HPAM + PYNPs)	0.90 − 1.0 − (0.2 + 0.60)	25.72
6	Na_2_CO_3_	PSC EOR 2.2	(HPAM + PYNPs)	0.90 − 0.98 − (0.2 + 0.60)	22.73
7	NaOH	PSC HOMF	(HPAM + CSNPs)	1.28 − 0.63 − (0.2 + 0.80)	35.0
8	NaOH	Mits-5L001	(HPAM + CSNPs)	1.28 − 1.0− (0.2 + 0.80)	23.15
9	NaOH	PSC EOR 2.2	(HPAM + CSNPs)	1.28 − 0.98 − (0.2 + 0.80)	25.22
10	Na_2_CO_3_	PSC HOMF	(HPAM + CSNPs)	0.90 − 0.63 − (0.2 + 0.80)	17.69
11	Na_2_CO_3_	Mits-5L001	(HPAM + CSNPs)	0.90 − 1.0 − (0.2 + 0.80)	27.56
12	Na_2_CO_3_	PSC EOR 2.2	(HPAM + CSNPs)	0.90 − 0.98 − (0.2 + 0.80)	21.66

**Table 6:** Buff Berea core sample characteristics.

Product ID	Formation	Permeability	Porosity	UCS	Steer by
SS-104	Upper Devonian	150–350 mD KCL 400–500 Md N_2_	20–22%	3800–4500 psi	KCL/N_2_

**Table 7:** Independent variables and their limits for optimum production of nanoparticles.

Acid Hydrolysis Parameters	Processability Ranges	
	Minimum	Maximum
Acid concentration, mol/L	2.2	3.6
Temperature, °C	40	60
Time, days	3	7

**Table 8:** Physical properties of NaOH and Na_2_CO_3_.

Property	Sodium Hydroxide	Sodium Carbonate
Purity	98%	99~100%
Molecular weight	39.997 g/mol	105.99 g/mol
Physical state	Solid	Powder
Odor	Odorless	Odorless
Color	Colorless	White
Melting temperature	323 °C	851 °C
Boiling temperature	1390 °C	1600 °C @ 760 mmHg
pH value	~14 at (50 g/L H_2_O, 20 °C)	-
Solubility in water	1090 g/L (at 20 °C)	22 g/100 mL (at 20 °C)
Solubility in ethanol	139 g/L	-
Specific gravity	-	2.53
Chemical formula	NaOH	Na_2_CO_3_

## Data Availability

Not applicable.

## Nomenclature

ASP	Alkaline–surfactant–polymer
Al_2_O_3_	Aluminum oxide
CAS	Cassava starch
CH_3_COOH	Acetic acid
CMC	Critical micelle concentration
cc	Cubic centimeter
cp	Centipoise
CS	Cassava starch
CSNF	Crystalline starch nanofluid
CSNPs	Starch nanoparticles
DSC	Differential scanning calorimetry
Dekasurf SF 9136	PT SPR Langgak special surfactant
EOR	Enhanced oil recovery
FTIR	Fourier transform infrared spectroscopy
Fsol	F solution
HPAM	Partially hydrolyzed polyacrylamide
IFT	Interfacial tension, mN/m
M	Mobility ratio of water to oil
Mits-5L001 PT SPR	Langgak special surfactant
NaOH	Sodium hydroxide
Na_2_CO_3_	Sodium carbonate
NPs	Nanoparticles
OOIP	Original oil in place
PV	Pore volume of sandstone core, cm^3^
ppm	Parts per million
PSA	Particle size distribution
PSC HOMF	PT SPR Langgak special surfactant
PSC EOR 2.2	PT SPR Langgak special surfactant
PYNPs	Purple yam nanoparticles
Q	Volumetric flow rate, cm^3^/min or ml/min
RF	Resistance factor
RFF	Residual resistance factor
Rf	Oil recovery percentage, %
SDBS	Sodium dodecylbenzene sulfonate
SDS	Sodium dodecyl sulfate
SFT	Surface tension, mN/m
SiO_2_	Silicon dioxide
S_or_	Residual oil saturation
TEM	Transmission electron microscopy
UV–VIS	Ultraviolet–visible spectrophotometer
ZnO	Zinc oxide
ΔP	Pressure difference, psi
ΔP_Polymer_	Differential pressure of polymer (or surfactant) injection, psi
ΔP_Brine_	Differential pressure of brine before polymer (or surfactant) injection, psi
ΔP_Brine after polymer injection_	ΔP of resumed brine flooding after polymer (or surfactant) injection, psi
